# Normal cell cycle progression requires negative regulation of E2F1 by Groucho during S phase and its relief at G2 phase

**DOI:** 10.1242/dev.201041

**Published:** 2023-06-01

**Authors:** Shaked Bar-Cohen, María Lorena Martínez Quiles, Alexey Baskin, Ruba Dawud, Barbara H. Jennings, Ze'ev Paroush

**Affiliations:** ^1^ Department of Developmental Biology and Cancer Research, Institute for Medical Research Israel-Canada, Faculty of Medicine, The Hebrew University, Jerusalem 91120, Israel; ^2^ Department of Biological and Medical Sciences, Oxford Brookes University, Oxford OX3 0BP, UK; ^3^ Department of Biological Chemistry, The Institute of Life Science, The Hebrew University of Jerusalem, Jerusalem 91904, Israel

**Keywords:** Cell cycle regulation, *Drosophila*, E2F1, Groucho, Protein phosphorylation, Repression

## Abstract

The cell cycle depends on a sequence of steps that are triggered and terminated via the synthesis and degradation of phase-specific transcripts and proteins. Although much is known about how stage-specific transcription is activated, less is understood about how inappropriate gene expression is suppressed. Here, we demonstrate that Groucho, the *Drosophila* orthologue of TLE1 and other related human transcriptional corepressors, regulates normal cell cycle progression *in vivo*. We show that, although Groucho is expressed throughout the cell cycle, its activity is selectively inactivated by phosphorylation, except in S phase when it negatively regulates E2F1. Constitutive Groucho activity, as well as its depletion and the consequent derepression of *e2f1*, cause cell cycle phenotypes. Our results suggest that Cdk1 contributes to phase-specific phosphorylation of Groucho *in vivo*. We propose that Groucho and its orthologues play a role in the metazoan cell cycle that may explain the links between TLE corepressors and several types of human cancer.

## Teaser

**Summary:** Groucho switches between active and inactive states during the cell cycle, and restricts gene expression in a phase-specific manner; relief of its repression is required for progression through G2 phase.

## INTRODUCTION

The cell cycle comprises a programmed sequence of events, including DNA synthesis, chromosome separation and cytokinesis. Progression through the cycle is highly regulated by protective checkpoints at which intrinsic or extrinsic conditions are monitored. The cycle arrests if suboptimal conditions are sensed, and recommences only once these are resolved through appropriate cellular response mechanisms (Clarke and Giménez-Abián, 2000; Kastan and Bartek, 2004; Musacchio and Salmon, 2007).

Cell cycle-related proteins are regulated at different levels. In the early *Drosophila* embryo, before the maternal-to-zygotic transition, rapid nuclear divisions are mainly controlled via translational and post-translational regulation of maternally-deposited determinants (Groisman et al., 2002; Becker et al., 2018). As zygotic transcription is induced, additional mechanisms are incorporated. For example, a surge of *string* (*cdc25*) expression is responsible for introducing the Gap 2 (G2) phase in otherwise Synthesis (S)-to-Mitosis (M) cycling embryonic cells (Edgar et al., 1994a; Budirahardja and Gönczy, 2009). Other genes that play a part in the cell cycle are also transcriptionally regulated, e.g., those involved in processes such as DNA replication and chromosome segregation, many of which are highly expressed in human tumours (Whitfield et al., 2002). Thus, transcriptional regulation in the context of the cell cycle is an important path to explore.

E2F1, a key cell cycle transcription factor, activates expression of *cyclin E* (*cycE*) and other target genes, the protein products of which are required at the initiation of S phase, and at the G2 and M phases (Bennett et al., 1996; Dimova et al., 2003; Dimova and Dyson, 2005; Herr et al., 2012). The E2F1 protein is detectable during all stages of the cell cycle except for S phase, when it is degraded (Reis and Edgar, 2004; Shibutani et al., 2008; Davidson and Duronio, 2012). Preventing E2F1 protein degradation during S phase leads to acceleration of the cell cycle and/or to apoptosis, highlighting the requirement for its removal at this stage (Shibutani et al., 2008). The activator functions of E2F1 and other cell cycle factors have been studied in depth; less is known, however, about transcriptional repressors in this process.

Transducin-like enhancer of split 1 (TLE1), a human orthologue of the *Drosophila* developmental corepressor Groucho (Gro), acts as an anti-proliferative factor (Zahavi et al., 2017), and TLE family members have been linked to human cancers (Kokabu et al., 2017; Dali et al., 2018). Furthermore, Gro and TLE1 are both phosphorylated in a cell cycle-regulated manner *in vitro* and in cultured cells (Nuthall et al., 2002), a modification previously shown to mitigate their corepressor activity downstream of receptor tyrosine kinase (RTK) pathways (Paroush et al., 1997; Hasson et al., 2005; Cinnamon et al., 2008; Helman et al., 2011, 2012; Johnston et al., 2016; Zahavi et al., 2017). Herein, we explore the possibility that Gro fulfils an *in vivo* regulatory function in the cell cycle.

We now demonstrate that Gro-mediated repression at S phase, and its relief at G2 phase, are both crucial for proper cell cycle progression. Specifically, we find that Gro is unphosphorylated and therefore active as a repressor only at S phase. We show that Gro binds within and around the *e2f1* gene locus, and that it is a negative regulator of its expression. *gro-*deficient cells display accelerated cell cycles and accumulate at Gap 1 (G1) phase, phenotypes resembling those caused by E2F1 overexpression. In addition, we find that overexpression of Gro causes cells to accumulate at G2 phase, and that Cyclin-dependent kinase 1 (Cdk1) normally phosphorylates Gro at this stage *in vivo*, suggesting that this mechanism attenuates the repressor activity of Gro and permits entry into mitosis. Together, our results reveal a novel role for Gro in the cell cycle, showing that it switches between active and inactive states and restricts gene expression in a phase-specific manner. We propose that a similar level of regulation underlies the involvement of TLE corepressors in cancer.

## RESULTS

### Groucho is selectively phosphorylated and inactive at mitosis and in the two Gap phases

Gro is uniformly expressed throughout *Drosophila* development (Delidakis et al., 1991). In any given nucleus, however, it is either primarily phosphorylated or mainly unmodified (Fig. S1) (Cinnamon et al., 2008; Helman et al., 2011; Johnston et al., 2016). Previous work demonstrated that phosphorylation of Gro, particularly by extracellular signal-regulated kinase (Erk) in response to RTK signalling, downregulates its repressor function (Hasson et al., 2005; Cinnamon et al., 2008; Helman et al., 2011, 2012). Thus, Gro has a regulated ability to switch between two modes: an active (unphosphorylated) or an inactive (phosphorylated) corepressor (Fig. S2).

**Fig. 1.**
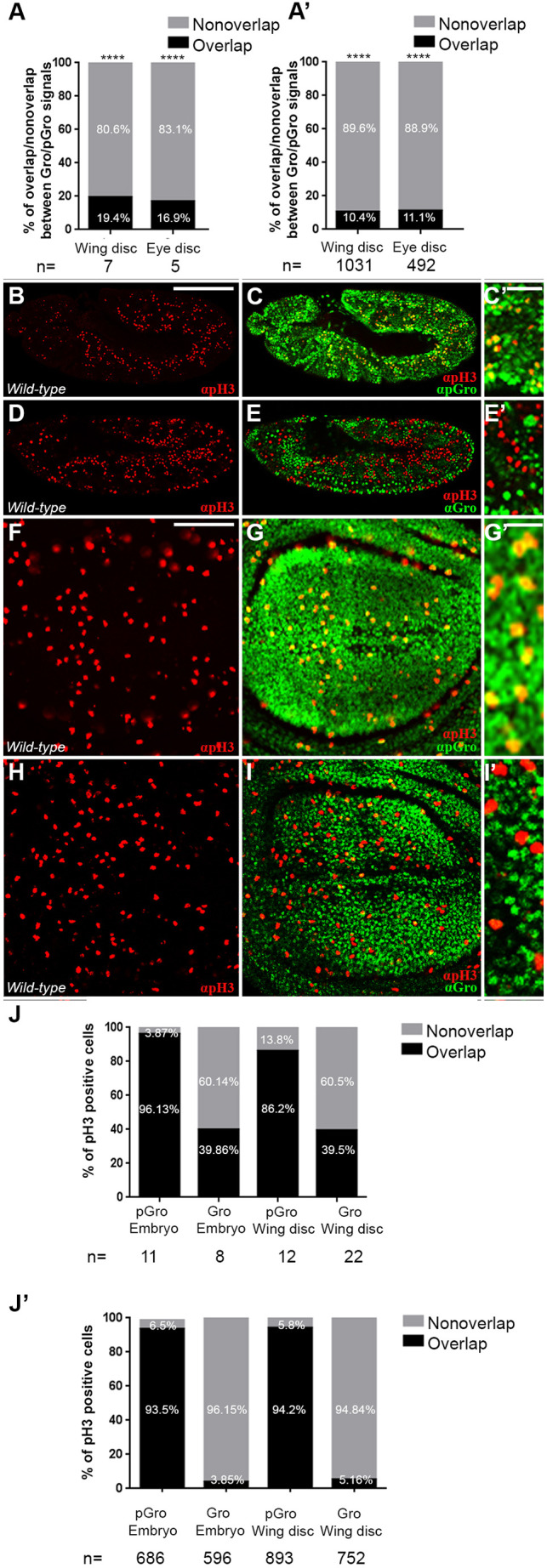
**Groucho is primarily phosphorylated in pH3-positive mitotic cells.** (A,A′) Quantification of the percentage of area (semi-automated; A) and the proportion of nuclei (manually scored; A′), co-stained (black) or not (grey) for both pGro and Gro in wing and eye imaginal discs (Fig. S1). (A) *n*=number of wing or eye imaginal discs scored in each case. (A′) *n*=number of nuclei scored in each case. ****P<0.0001 (binomial test, based on previous studies showing that these signals do not overlap in the embryo). (B-I′) Confocal images of stage 11 wild-type embryos (lateral views; B-E′) and wing imaginal discs from wild-type third instar wandering larvae (F-I′), co-stained for pH3 (red; B-I′) together with either pGro (green; C,C′,G,G′) or Gro (green; E,E′,I,I′). (C′,E′,G′,I′) Magnified views of C,E,G and I, respectively. (J,J′) Quantification of the percentage of pH3-positive area (semi-automated; J), and of the percentage of pH3-positive nuclei (manually scored; J′), co-stained (black) or not (grey) for pGro or for Gro, in embryos (two left columns) and in wing imaginal discs (two right columns). (J) *n*=number of embryos or wing imaginal discs scored in each case. (J′) *n*=number of pH3-positive cells scored in each case. Scale bars: 100 µm (B-I); 16.6 µm (C′,E′,G′,I′).

Double staining of cycling cells in imaginal discs, with antibodies that are largely specific for Gro and for phosphorylated Gro (pGro; Materials and Methods), revealed that they display limited overlap in their respective signals. In both wing and eye imaginal discs, only 10-20% of cells showed overlapping nuclear signals for both antibodies (Fig. 1A,A′; Fig. S1) (Cinnamon et al., 2008; Helman et al., 2011; Johnston et al., 2016). Using these two antisera, we show below that phosphorylation of Gro fluctuates dynamically in a cell cycle phase-dependent manner, and that it is predominantly modified at all stages of the cell cycle except for S phase.

Specifically, mitotic cells, distinguishable by anti-phospho-Histone 3 at Serine 10 (pH3) staining, were generally positive for pGro, both in embryos (Fig. 1B-C′) as well as in wing imaginal discs (Fig. 1F-G′,J,J′). In contrast, the pH3 signal complemented that of the unphosphorylated active form of Gro in both tissues (Fig. 1D-E′,H-I′,J,J′), indicating that Gro is largely phosphorylated, and therefore inactive as a repressor, at mitosis.

**Fig. 2.**
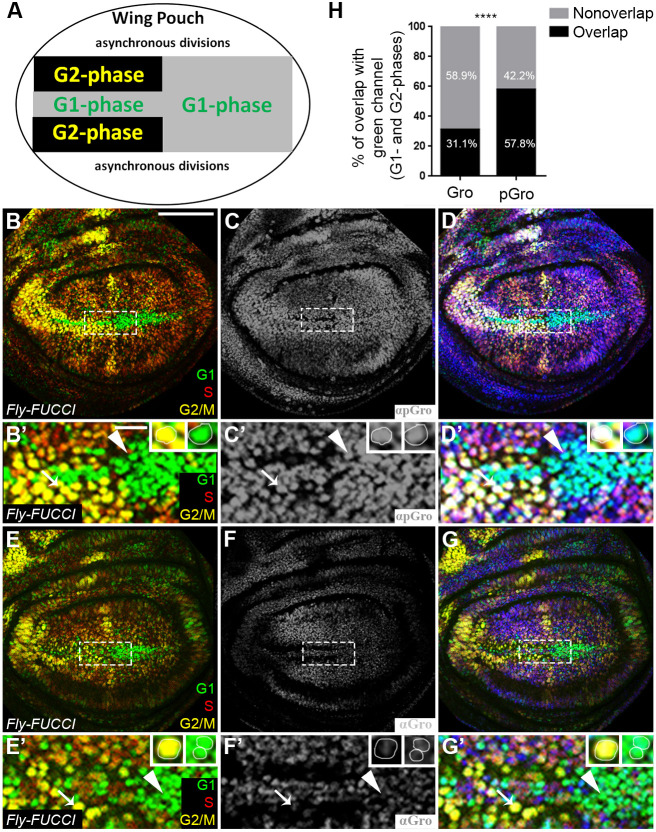
**Groucho is phosphorylated during G1 , G2 and M phases.** (A) Schematic representation of the central part of the wing imaginal disc (area boxed in B-G) (adapted from Zielke et al., 2014). In this region, a stripe of anterior cells that are arrested at the G1 phase (grey) are flanked by cells arrested at the G2 phase (black), and cells in the posterior domain are arrested at G1 phase. (B-G′) Confocal images of *Fly-FUCCI* third instar wandering larval wing imaginal discs, stained for pGro (grey in C,C′; blue in D,D′) or for Gro (grey in F,F′; blue in G,G′). In this system, the S-phase cell population is in red, cells at G2/M phases are stained yellow and those at the G1 phase are in green (B,B′,D-E′,G,G′). (B′,C′,D′,E′,F′,G′) Magnified views of the central (boxed) region of the wing imaginal disc blade shown in B,C,D,E,F,G, respectively. Insets in panels of magnified views show representative cells either in G2/M phase (yellow; left) or in G1 phase (green; right). Note that pGro staining is evident in G2/M-phase nuclei, as well as in cells at G1 phase (arrow and arrowhead, respectively, in B′,C′,D′), but that of Gro is undetectable in these cells (arrow and arrowhead in E′,F′,G′, respectively). The red S-phase marker alone was not analysed due to intensity ambiguity. (H) Semi-automated quantification of the percentage of the area covered by cells in G1 and G2 phases (green), co-stained (black) or not (grey) with anti-Gro (left) or with anti-pGro (right) in wing imaginal discs. Nine discs were quantified for pGro and ten for Gro. ****P<0.0001 (two-tailed *t*-test). Scale bars: 100 µm (B-G); 16.6 µm (B′-G′).

Not all pGro-positive nuclei were mitotically active, however (Fig. 1C′,G′); Gro was also phosphorylated at the G1 and G2 phases as determined using *Fly-FUCCI*, an *in vivo* fluorescent, ubiquitination-based indicator system (Fig. 2) (Zielke et al., 2014). In *Fly-FUCCI* wing imaginal discs, individual cells appear in different colours according to their cell cycle stage (Fig. 2A-B′,E-E′). Staining with anti-pGro and anti-Gro antibodies demonstrated that Gro is mostly phosphorylated at the G2/M and G1 phases in asynchronously-dividing cells throughout the wing imaginal disc. This was best seen in cells along the prospective dorsoventral boundary of the wing pouch (Fig. 2A), where whole populations of cells reduce their proliferation rate and are found either at the G1 or G2 phase of the cycle (Fig. 2B-G′,H). We surmise that Gro is extensively phosphorylated and, consequently, mostly inactive at M, G1 and G2 phases.

### Groucho is unphosphorylated, and therefore an active repressor, at S phase

**Fig. 3.**
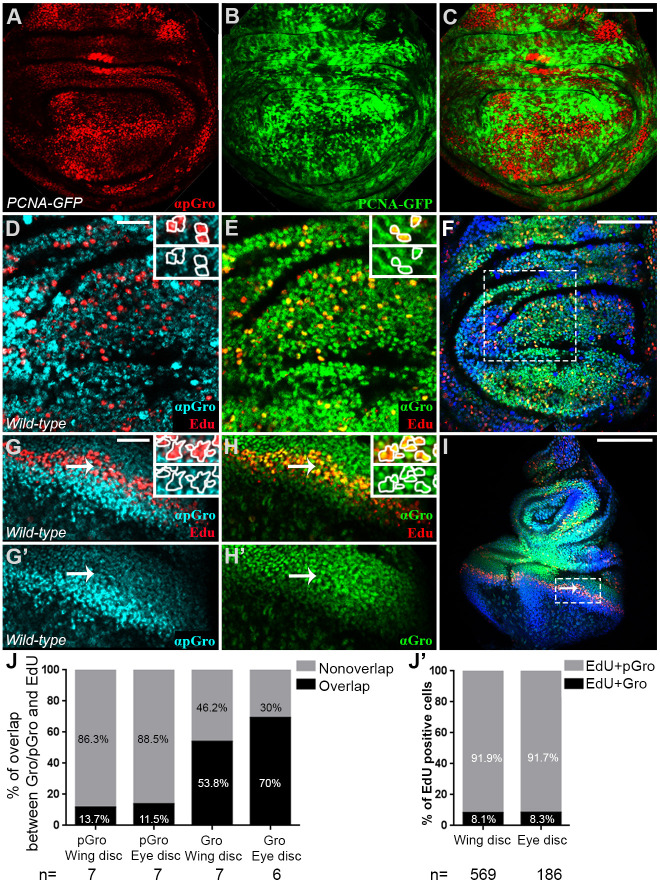
**Groucho is unphosphorylated during S phase.** (A-C) Confocal image of a *PCNA-GFP* third instar wandering larval wing imaginal disc, stained for pGro (red; A,C). Cells in S phase are GFP-positive (green; B,C). (D-I) Confocal images of third instar wild-type wandering larval wing (D-F) and eye (G-I) imaginal discs, stained for pGro (turquoise in D,G,G′; blue in F,I), Gro (green; E,F,H-I) and EdU (red; D-I). (D,E,G-H′) Magnified views of the boxed regions in F and I, respectively. Arrows (G-I) point at the stripe of EdU-positive, S-phase cells posterior to the morphogenetic furrow. Insets in (D,E,G,H) show magnified views of individual cells stained either for pGro and EdU (D,G) or for Gro and EdU (E,H). (J) Semi-automated quantification of the percentage of EdU-positive area, co-stained (black) or not (grey) for pGro or for Gro in wing imaginal discs (two left columns) and in eye imaginal discs (two right columns). *n*=number of imaginal discs scored in each case. (J′) Percentage of manually scored EdU-positive nuclei, co-stained for Gro (black) or for pGro (grey) in wing and eye imaginal discs. *n*=number of EdU-positive cells scored in each case. Scale bars: 100 µm (A-C,F); 33.3 µm (D,E,G-H′); 200 µm (I).

The *Fly-FUCCI* system also indicated that Gro is unphosphorylated at S phase. To confirm this, we carried out two additional experiments. First, wing imaginal discs of *PCNA-GFP* flies, in which expression of cytoplasmic GFP is a reliable marker for early S phase (Thacker et al., 2003; Strzalka and Ziemienowicz, 2011), were stained for pGro. As Fig. 3A-C shows, the pattern of GFP largely complemented that of pGro. Second, we stained wing imaginal discs with the nucleoside thymidine analogue 5-ethynyl-2′-deoxyuridine (EdU), which labels cells at late S phase (Buck et al., 2008). Here, too, we found that EdU-positive cells mainly co-stain for unphosphorylated Gro but not for pGro (Fig. 3D-F; J-J′).

A similar result is also observed in eye imaginal discs, which allow the analysis of a relatively synchronised S phase cell population. In this tissue, differentiation proceeds as a wave across the disc such that a stereotypic stripe of undifferentiated cells, located posteriorly to the morphogenetic furrow, synchronously enter S phase (arrows; Fig. 3G-I) (Wolff and Ready, 1991). The vast majority of these EdU-positive S-phase cells stained for Gro but not for pGro (Fig. 3G-H′,J,J′), leading us to conclude that, in cycling cells, Gro is unphosphorylated only at S phase. Its repressive activity is, therefore, restricted to this specific stage of the cell cycle.

### Ectopic expression of Groucho reduces the number of mitotic cells

The roughly non-overlapping patterns of anti-pH3 and anti-Gro staining (Fig. 1) suggest that Gro is primarily phosphorylated in mitotic cells, and therefore inactive at this stage. To determine the significance of Gro phosphorylation to mitosis, we assessed the effects of expressing a non-phosphorylatable Gro mutant on the number of mitotic cells in the rapidly dividing wing imaginal disc. Towards this end, we employed a non-phosphorylatable Gro variant mutated in its two phosphoacceptor sites (Gro^AA^), in addition to a phosphomimetic Gro mutant derivative (Gro^DD^) (Hasson et al., 2005). If phosphorylation of Gro is a precondition for mitosis, we expect the expression of the constitutively active Gro^AA^ repressor to dominantly reduce the pH3 signal, but not expression of Gro^DD^, the repressor activity of which is attenuated (although recognised by anti-panTLE antibodies, we cannot formally rule out the possibility that Gro^DD^ is partially inactive due to misfolding; Fig. S3) (Hasson et al., 2005; Cinnamon et al., 2008; Helman et al., 2011, 2012; Johnston et al., 2016).

**Fig. 4.**
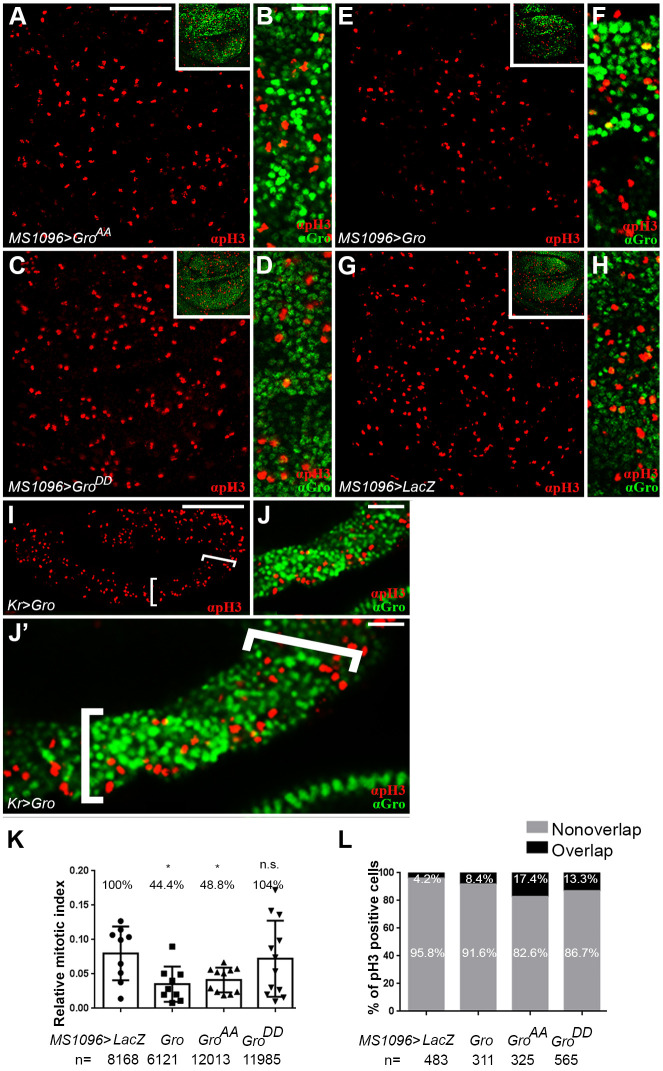
**Ectopic expression of Groucho reduces the number of pH3-positive cells.** (A-J′) Confocal images of wing imaginal discs (A-H) and stage 11 embryo (lateral view; I-J′), ectopically expressing non-phosphorylatable Gro (Gro^AA^; A,B), phosphomimetic Gro (Gro^DD^; C,D) or native Gro (E,F,I-J′). (G,H) *lacZ*-expressing control. Embryos and imaginal discs were co-stained for pH3 (red; A-J′) and for Gro (green; B,D,F,H,J,J′). (I,J′) The *Kr>Gal4* expression domain is delineated by brackets. (B,D,F,H,J,J′) Magnified views of cells in panels A,C,E,G,I, respectively. Insets in A,C,E,G show that ectopic expression of either Gro^AA^ (A) or Gro (E) masks the detection of endogenous Gro by the anti-Gro antibody, and that this anti-Gro antibody does not recognise ectopically-expressed Gro^DD^ (C) due to its specificity towards unphosphorylated Gro. Hence, endogenous Gro is only observed in discs expressing Gro^DD^ (C) or *lacZ* (G) (Fig. S3; Materials and Methods). (B,F,J,J′) Patchy *Gal4*-driven expression leads to uneven Gro protein levels (Fig. S3). (K) Graph showing relative mitotic indices, quantified based on the ratio of pH3-positive cells relative to the number of total nuclei marked by DAPI staining. Each dot in the graph represents the relative mitotic index measured in a single wing imaginal disc (nine discs were analysed for *lacZ*, nine for Gro, 11 for Gro^AA^ and 12 for Gro^DD^). *n*=number of nuclei scored in each case. *P<0.05 mitotic indices of Gro and Gro^AA^ compared with that of *lacZ* (Mann–Whitney U-test). The mitotic index of Gro^DD^ compared with that of *lacZ* is non-significant (n.s.). In all cases, data represent the mean±s.d. The calculated mitotic index in each case is presented as percentage relative to the *lacZ* index (given a value of 100%). (L) Percentage of pH3-positive nuclei, coinciding (black) or not (grey) with Gro staining in the indicated wing imaginal discs. *n*=number of pH3-positive cells scored in each case. Scale bars: 100 µm (A,C,E,G,I); 16.6 µm (B,D,F,H); 50 µm (J); 33.3 µm (J′).

The two variants were ectopically expressed in wing imaginal discs under Gal4/UAS control (Brand and Perrimon, 1993). Expression of Gro^AA^ using an early driver (*nubbin-Gal4*) resulted in small dysmorphic discs, preventing their subsequent analysis. Instead, we used *MS1096-Gal4*, which drives non-uniform expression relatively late and mainly in the dorsal wing compartment (Fig. S3) (Milán et al., 1998). In this case, expression of Gro^AA^, but not of the phosphomimetic Gro^DD^ variant, reduced the number of pH3-positive cells compared with control *lacZ*-expressing wings (Fig. 4A-D,G,H), and the ensuing adult wings were markedly smaller (Fig. S3). These results suggested that phosphorylation of Gro correlates with progression to mitosis [other, unrelated functions of Gro in imaginal disc development probably also contribute to the severe adult wing phenotypes brought about by its overexpression (Cavallo et al., 1998; Hasson et al., 2001, 2005)]. There was also a significantly greater overlay between the pH3 and Gro^DD^ signals than between those of pH3 and Gro^AA^ (Fig. S3), indicating that cells expressing Gro^DD^, but not those expressing Gro^AA^, more readily enter mitosis. Phosphorylated Gro is, therefore, compatible with M phase.

Induced expression of native Gro also caused a significant reduction in the number of pH3-positive mitotic cells, both in wing imaginal discs (Fig. 4E-H) as well as in the embryo, which represents a different developmental tissue [using the *Krüppel* (*Kr*)-*Gal4* driver; Fig. 4I-J′] (Chu et al., 1998). To quantify Gro's effects on mitosis, while controlling for changes in cell size exerted by its expression, we calculated the ratio between the number of pH3-positive cells and the total number of cells in the wing pouch region (mitotic index). We found that the mitotic indices of wing imaginal discs overexpressing Gro (44.4%) and Gro^AA^ (48.8%), but not Gro^DD^ (104%), were significantly lower than the mitotic index observed in control *lacZ*-expressing discs (related to as 100%; Fig. 4K). Notably, expression of native transcriptional regulators and their non-phosphorylatable derivatives often exerts similar effects in other biological settings [e.g. figure 4 in Cinnamon et al. (2008); figures 3D-F and 4C in Li et al. (2016) and figure 5A,B in Lee et al. (2010)].

Strikingly, irregular Gal4-driven expression (Fig. S3) (Ward et al., 2002; Barth et al., 2012) revealed that the negative effect of Gro^AA^ and Gro on mitosis is largely cell-autonomous: most of the remaining pH3-positive cells in the *MS1096-* and *Kr-Gal4* expression domains were consistently those that expressed null or low levels of induced Gro^AA^ and Gro (Fig. 4B,F,L; Movie 1). The discordance between the anti-Gro and -pH3 signals, which is also largely apparent in wing discs overexpressing Gro^DD^ and *lacZ* (Fig. 4D,H), is consistent with the idea that entry into M phase necessitates the previous attenuation of Gro repressive activity by phosphorylation.

### Cdk1 phosphorylates Groucho *in vivo*

Given that phosphorylation of Gro fluctuates dynamically in a cell cycle phase-dependent manner, Gro must be phosphorylated by a kinase that is active at G2 phase but inactive at S phase. Although Erk activity impinges on cell cycle regulation at different levels (Prober and Edgar, 2000; Mogila et al., 2006; Mirzoyan et al., 2019), there is no evidence that it exhibits similar activation and inactivation dynamics. Instead, we considered Cdk1 as the prime candidate for phosphorylating Gro in this context (Vidwans and Su, 2001). Cdk1 activity shows the appropriate dynamics for regulating Gro activity, being essential at G2 and M phases but inactive at S phase (Bettencourt-Dias et al., 2004). In addition, Cdk1 phosphorylates Gro and its human TLE1 orthologue *in vitro* and in cell culture (Nuthall et al., 2002). Finally, Cdk1 is a proline-directed serine/threonine kinase (Malumbres, 2014) that, like Erk, is expected to target the Gro proline-glycine-threonine-proline motif. Indeed, the Gro Cdk1 phosphorylation site was previously mapped to a fragment encompassing the Erk phosphorylation motif recognised by the anti-pGro antibodies (Materials and Methods) (Nuthall et al., 2002).

**Fig. 5.**
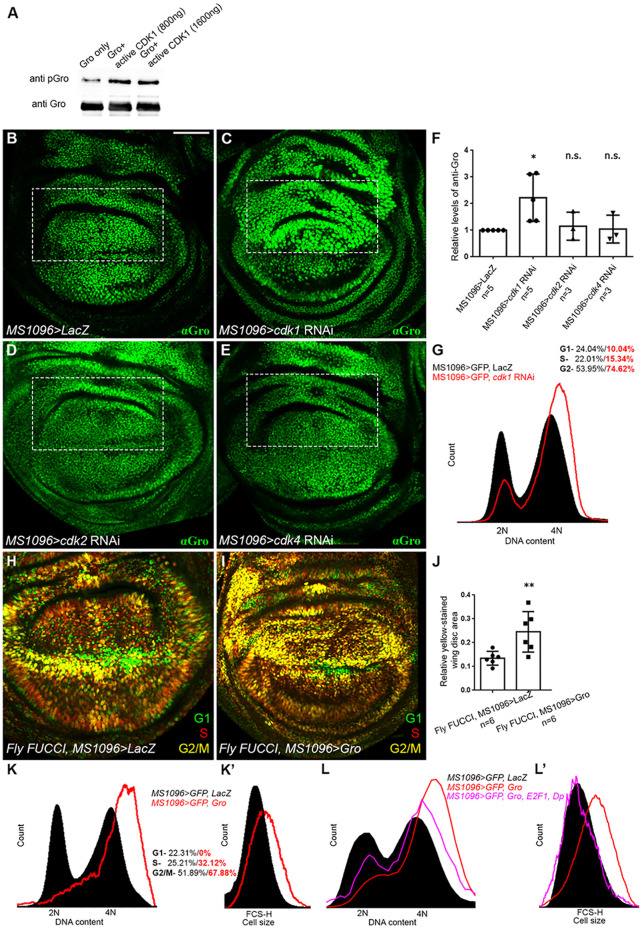
**Cells accumulate at G2 phase upon ectopic expression of Groucho.** (A) An activated Cdk1/CycB complex phosphorylates GST-tagged, full-length Gro *in vitro*. Three independent kinase assays resulted in similar outcomes. (B-F) Cdk1 phosphorylates Groucho *in vivo*. (B-E) Confocal images of third instar wandering larval wing imaginal discs expressing either *lacZ* (B) or RNAi constructs for *cdk1* (C), *cdk2* (D) or *cdk4* (E), stained for Gro (green). Two RNAi lines, targeting each Cdk, produced similar outcomes. The boxed regions demarcate the predominantly dorsal expression domain of the *MS1096-Gal4* driver (Fig. S3). Note that *RNAi*-based knockdown of *cdk1* (C), but not of *cdk2* (D) or *cdk4* (E), leads to the accumulation of unphosphorylated Gro (see *lacZ*-expressing disc; B). (F) Graph showing relative Gro protein levels determined by western blot analyses of whole wing imaginal disc lysates from the indicated genetic backgrounds, immunoblotted with anti-Gro and anti-Actin antibodies. Relative Gro levels were determined based on the ratio between Gro and Actin, normalised to that in *lacZ*-expressing controls. The fold increase in the level of unphosphorylated Gro upon *cdk1* knockdown (2.222±0.3975) is not observed in *cdk2* or *cdk4* knockdowns (1.152±0.3023 and 1.045±0.3030, respectively); *P<0.05 for *cdk1* RNAi compared with *lacZ* control; non-significant (n.s.) for *cdk2* and *cdk4* RNAi compared with *lacZ* control (Mann–Whitney U-test). *n*=number of biological repeats conducted for each genotype. In all cases, data represent the mean±s.d. The increase in the level of unphosphorylated Gro following *cdk1* knockdown is probably a gross underestimate, given that *MS1096-Gal4* drives non-uniform expression in only a subset of cells in the wing imaginal disc (Fig. S3). (G) Cell cycle distribution of GFP-positive cells, dissociated from larval wing imaginal discs co-expressing either GFP together with *lacZ* (black) or along with *cdk1* RNAi (red contour) under the *MS1096-Gal4* driver. DNA content was determined using Hoechst 33342 and normalised to number of events. (H,I) Confocal images of wing imaginal discs, dissected from *Fly-FUCCI* third instar wandering larvae expressing either *lacZ* (H) or Gro (I) under *MS1096-Gal4* regulation. (J) The enrichment of yellow-stained, G2/M-phase cells following Gro overexpression, relative to *lacZ*-expressing controls, was quantified by delimiting the wing pouch regions and then measuring levels of yellow colour coverage (restricted to the yellow channel; Adobe Photoshop) in the selected area using ImageJ. Graph shows the relative area of yellow-stained *Fly-FUCCI* cells in *lacZ-* (left) or Gro-expressing (right) wing imaginal discs, under the regulation of *MS1096* driver. *n*=number of wing discs scored in each case. **P<0.01 (Mann–Whitney U-test). In all cases, data represent the mean±s.d. (K,K′) Flow cytometric analyses of GFP-positive cells, dissociated from wing imaginal discs of flies expressing GFP together with *lacZ* (black) or GFP along with Gro (red contour), under the regulation of the *MS1096-Gal4* driver. The DNA content was determined using Hoechst 33342 and normalised to number of events. (K) Cell cycle distribution of GFP-positive *lacZ*-expressing cells or of GFP-positive Gro-expressing cells is depicted as percentages in black and red, respectively. The number of cells at G2/M phases, following Gro misexpression, increases. (K′) Forward scatter-height (FSC-H) from the same experiment, showing that the relative cell size in the Gro-expressing population (red contour) is generally larger than that of cells in the control population (black). (L,L′) Cell cycle distribution (L) and FSC-H reflecting cell size (L′) of GFP-positive cells, dissociated from larval wing imaginal discs co-expressing either GFP together with *lacZ* (black); GFP together with Gro alone (red contour); or GFP together with Gro, E2F1 and Dp (pink contour) under the *MS1096-Gal4* driver. DNA content was determined using Hoechst 33342 and normalised to number of events. Scale bar: 100 µm (B-E,H,I).

We first confirmed, biochemically, that a purified, activated Cdk1/CyclinB1 complex can phosphorylate recombinant Gro protein *in vitro*. We found that Cdk1 phosphorylates threonine 308, the same phosphoacceptor amino acid that is modified by Erk and recognised by anti-pGro antisera (Fig. 5A). To test whether Gro also undergoes phosphorylation by Cdk1 *in vivo*, RNA interference (RNAi) was used to deplete it. Expression of *cdk1* RNAi in wing imaginal discs resulted in fewer and larger cells, a known phenotype caused by Cdk1 deficiency (Fig. S4) (Weigmann et al., 1997; Johnston, 1998; Bettencourt-Dias et al., 2004). As Fig. 5 shows, the amount of unphosphorylated Gro increases in such *MS1096*>*cdk1* RNAi wing imaginal discs, predominantly in the dorsal compartment (Fig. 5B-F) (the possibility that phosphorylation by Cdk1 also affects Gro protein stability cannot be ruled out) (Milán et al., 1998). Conversely, the pGro signal decreases in *cdk1* RNAi wing imaginal discs, although, due to high variability, this reduction is statistically non-significant (Fig. S4). A comparison between the anterior and posterior compartments of *en*>*cdk1* RNAi-expressing wing imaginal discs, however, disclosed a significant reduction in the pGro signal upon Cdk1 knockdown (Fig. S5).

The observed increase in unphosphorylated Gro is not simply due to a G2/M phase arrest caused by *cdk1* depletion (Fig. 5G) (Bettencourt-Dias et al., 2004), as Gro is normally phosphorylated at these stages of the cell cycle (Fig. 2). Our results are, therefore, consistent with Cdk1 being the kinase responsible for inactivating Gro during G2 phase *in vivo*. Noteworthy, Gro is predominantly phosphorylated at G1 phase when Cdk1 is inactive, possibly due to the persistence of its phosphorylation state (Cinnamon et al., 2008; Helman et al., 2011) or via some other kinase(s).

### Phosphorylation of Groucho correlates with progression into mitosis

The Cdk1-mediated phosphorylation of Gro at the exit from S phase appears to be required for normal cell cycle progression. When Gro is overexpressed in *Fly-FUCCI* wing imaginal discs, a substantial enrichment in yellow-stained, G2/M-phase cells relative to control discs occurs (Fig. 5H-J). As similar Gro expression leads to a cell-autonomous reduction in pH3 staining (Fig. 4), we concluded that the yellow staining reflects a significant increase in the number of cells at G2 phase.

To confirm this result, we conducted flow cytometric analysis of dissociated wing imaginal disc cells. As Fig. 5K shows, a higher proportion of cells ectopically expressing Gro are in G2/M phases compared with cells from control wings. Gro-expressing cells are also larger in size than control cells (Fig. 5K′) (Bettencourt-Dias et al., 2004). These results, together with the largely overlapping expression patterns of pH3 and pGro (but not Gro) staining (Fig. 1) and the Gro-induced decline in pH3-positive mitotic cells (Fig. 4), indicate that cells overexpressing Gro accumulate at G2 phase, before mitosis, leading us to conclude that unphosphorylated, repressive Gro hinders normal cell cycle progression.

### Groucho negatively regulates *e2f1* expression

An *in silico* approach was used to identify potential cell cycle targets of Gro-mediated repression. A list of genes was first compiled based on their association with the terms ‘cell cycle’ and/or ‘cell proliferation’. This list was then intersected with lists of genes that are located in proximity to Gro peaks, drawn from various genome-wide studies that profiled Gro binding to chromatin (Orian et al., 2007; Shibutani et al., 2008; Roy et al., 2010; Kaul et al., 2014; Chambers et al., 2017). Candidate Gro-regulated target genes were then ranked based on the number of lists they appear in (Table S1).

From this gene set we chose, as case in point, the *e2f1* gene for further investigation. *e2f1* stands out as an attractive Gro target, as it encodes a key cell cycle regulator (Asano et al., 1996; Neufeld et al., 1998). The E2F1 protein is detectable at all phases of the cell cycle except for S phase, when it is degraded (Bennett et al., 1996; Dimova et al., 2003; Davidson and Duronio, 2012; Herr et al., 2012). The inappropriate presence of E2F1 at S phase can lead to accelerated cell cycle and/or apoptosis (Bennett et al., 1996; Dimova et al., 2003; Davidson and Duronio, 2012; Herr et al., 2012). Although post-translational regulation of E2F1 has been extensively studied, regulation of *e2f1* gene expression is, as yet, largely unexplored (Johnson et al., 1994; Goulev et al., 2008; Øvrebø and Bradley-Gill, 2022).

**Fig. 6.**
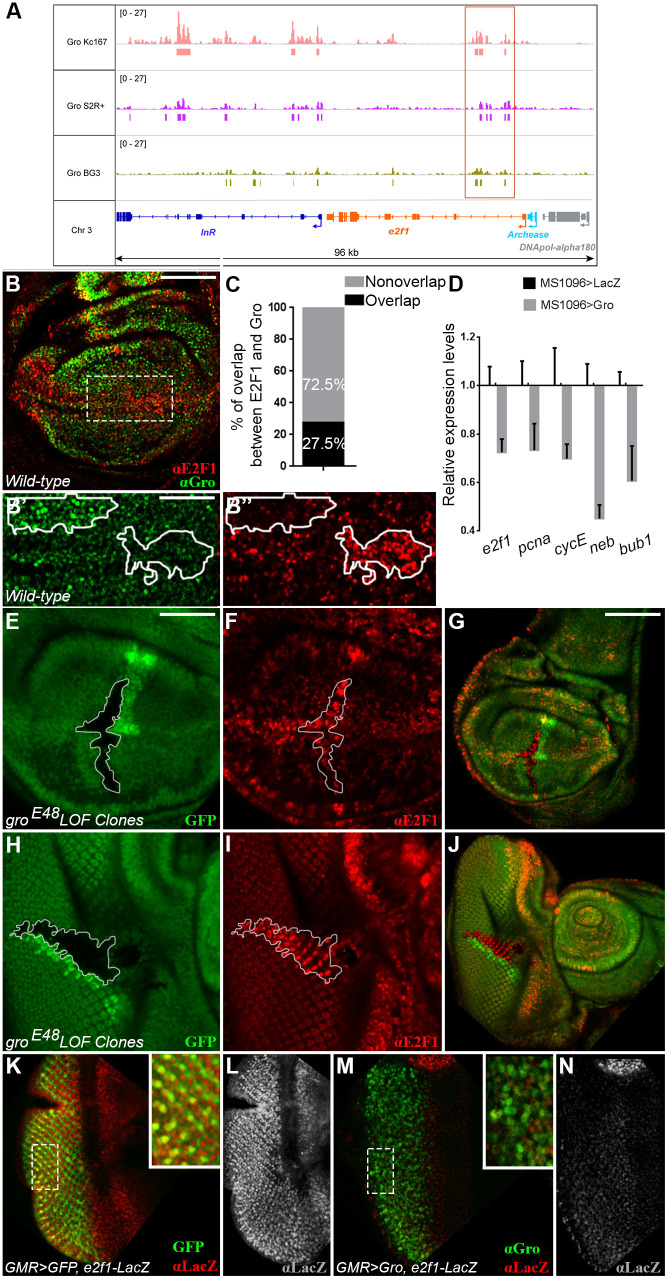
**Groucho represses *e2f1* expression.** (A) Gro binds in shared clusters within (boxed) and downstream of the *e2f1* gene locus in three *Drosophila* cell lines, derived from different origins. Panel shows Genome Browser view of ChIP-seq data analyses depicting the profiles of Gro binding in Kc167, S2R+ and BG3 cells. ChIP-seq signals are quantified as counts per million. Significant peaks of Gro binding are marked as bars under the ChIP-seq tracks in each cell line (typically FDR≤10%; Materials and Methods). (B-B″) Confocal image of wild-type third instar wandering larval wing imaginal disc, co-stained for E2F1 (red) and Gro (green). B′ and B″ show magnified views of the boxed region in B. (C) Semi-automated quantification of the percentage of area co-stained for E2F1 and Gro in 12 wing imaginal discs. (D) RT-PCR analyses of mRNA extracted from third instar wandering larval wing imaginal discs expressing either Gro (grey) or *lacZ* (black) under *MS1096-Gal4* regulation. Relative transcript levels of *e2f1* and its targets *pcna*, *cycE*, *neb* and *bub1* are reduced in Gro-expressing discs, normalised to *lacZ* controls. Gro does not bind in proximity to the *pcna* and *cycE* loci, and *neb* and *bub1* each appears in a single gene set; therefore, Gro probably affects their expression levels indirectly, via repression of *e2f1*. The ∼30% reduction in *e2f1* levels is probably an underestimation, given the mosaic expression driven by *MS1096>Gal4* (Fig. S3). Data represent the mean±s.d. (E-J) Homozygous *gro^E48^* loss-of-function clones (demarcated by white contours in E,F,H,I), discernible as GFP-negative and accompanied by GFP-positive twin spot clones (green; E,G,H,J), were induced in larval wing (E-G) and eye (H-J) imaginal discs. E,F,H and I show magnified views of clones in G and J, respectively. E2F1 (red; F,G,I,J) is derepressed and ectopically accumulates in *gro* mutant clones. (K-N) Confocal images of third instar wandering larval eye imaginal discs, in which *GMR-Gal4* drives the expression of either GFP (green; K) or of Gro (green; M), co-stained for *e2f1-lacZ* reporter expression (*lacZ*; red in K,M; grey in L,N). Insets show magnified views of the boxed regions in K and M, respectively. (K) *GMR-Gal4* drives expression of GFP (green) in differentiating retinal neurons (Yeates et al., 2019). These cells also express the *lacZ* reporter gene (red) derived from the *e2f1-lacZ* enhancer trap. (M) Gro expression causes an overall reduction in anti-*lacZ* staining (red), particularly in the retinal neuronal cells overexpressing Gro (green). Scale bars: 100 µm (B,E,F,K-N); 50 µm (B′,B″,H,I); 200 µm (G,J).

We confirmed Gro binding to the *e2f1* locus by performing chromatin immunoprecipitation assays with sequencing (ChIP-seq) using ML-DmBG3-c2 (BG3) cells, which originate from the larval central nervous system (Cherbas et al., 2011). We also re-examined ChIP-seq data previously generated from two embryonic plasmatocyte-derived cell lines, Kc167 and S2R+ (Kaul et al., 2014). In all three cell lines, we found a shared cluster of Gro peaks within the *e2f1* gene that maps to the first intron of the *e2f1-RA* and *e2f1-RB* transcripts, and is upstream of the transcription start sites of four other *e2f1* transcripts (RC, RD, RE and RF) (Fig. 6A; boxed). Other common peaks of Gro binding are found just downstream of the *e2f1* gene (Fig. 6A). The widespread recruitment of Gro in and around the *e2f1* locus in varied cell types, derived from different developmental stages, is consistent with *e2f1* being a general, rather than a cell-type specific, Gro target.

Several analyses support the notion that Gro represses *e2f1* gene expression during S phase, and that phosphorylation of Gro attenuates this repression. Immunofluorescence staining of wild-type wing imaginal discs shows that signals of E2F1 and non-phosphorylated repressive Gro do not greatly overlap (Fig. 6B,C). In addition, ectopic Gro^AA^ expression significantly reduces anti-E2F1 staining**,** whereas Gro^DD^ expression does not affect E2F1 levels or distribution (Fig. S6). Moreover, real-time polymerase chain reaction (RT-PCR) assays revealed that overexpression of Gro in wing imaginal discs decreased the relative transcript levels of *e2f1* and several of its targets (Fig. 6D). Importantly, E2F1 is derepressed in homozygous *gro^E48^* mutant clones, induced in either wing (Fig. 6E-G) or eye imaginal discs (Fig. 6H-J). Finally, we used an *e2f1-lacZ* enhancer-trap line to distinguish between a role for Gro in the transcriptional regulation of the *e2f1* gene or in the post-translational control of E2F1 protein levels (Duronio et al., 1995; Brook et al., 1996). As shown in Fig. 6K-N, Gro negatively regulates reporter expression derived from this enhancer-trap in eye imaginal discs, indicating that, directly or indirectly, it represses *e2f1* transcription.

We reasoned that if the cell accumulation at G2 phase, induced by overexpression of Gro, involves the inappropriate repression of *e2f1* at G2 phase, a stage when it should be re-transcribed, then the concomitant expression of *e2f1* will rescue this phenotype. As Fig. 5L,L′ shows, the co-expression of E2F1 and Dimerization partner (Dp) suppresses the G2-phase accumulation prompted by Gro overexpression, and cell size reverts to normal. This functional link between Gro and E2F1 reinforces the notion that relief of Gro-mediated *e2f1* repression at G2 phase, via Cdk1-dependent phosphorylation, is crucial.

### Gro-mediated repression at S phase is required for normal cell cycle progression

**Fig. 7.**
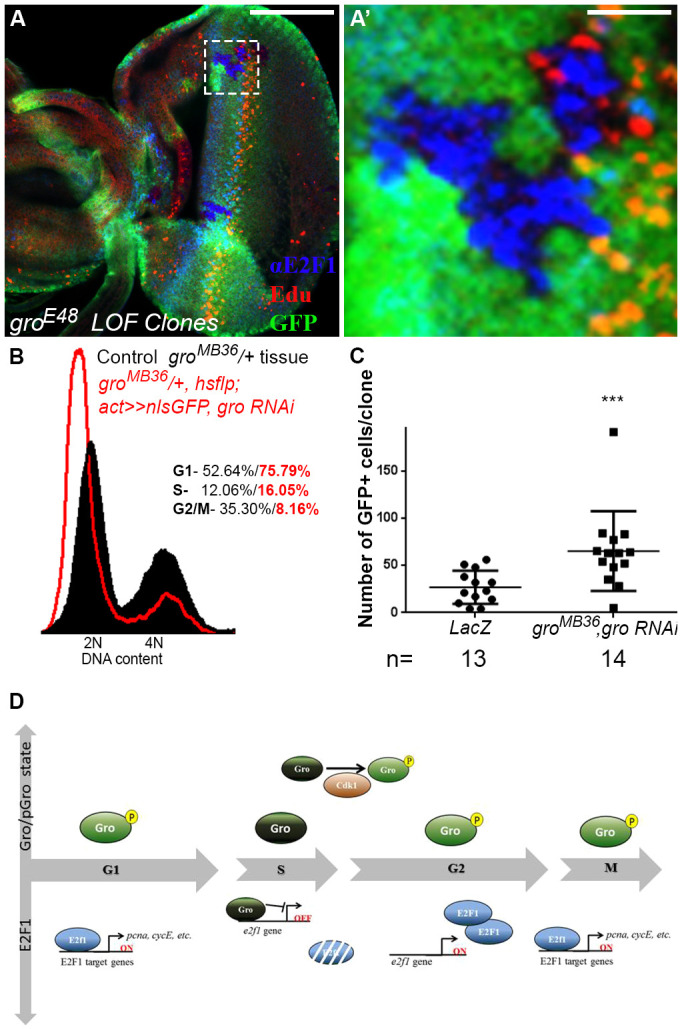
**Cells devoid of *groucho* undergo accelerated cell cycles and accumulate at G1 phase.** (A,A′) Confocal images of third instar wandering larval eye imaginal disc, stained for E2F1 (blue) and EdU (red). *gro* clones are detectable by lack of GFP staining and by adjacent GFP-positive twin spot clones (green). A′ shows magnification of boxed region in A, focusing on a *gro* mutant clone overlapping the morphogenetic furrow. Strikingly, all *gro* mutant cells that accumulate E2F1 do not stain for EdU and are, therefore, not in S phase. (B) Flow cytometric analyses of dissociated cells from eye imaginal discs. *gro^MB36^*/+ cells expressing *gro* RNAi are labelled with GFP (red contour), whereas *gro^MB36^/+* cells that do not express *gro* RNAi are GFP-negative (black). The DNA content was determined using Hoechst 33342, and normalised to number of events. The cell cycle distribution of GFP-positive cells in which *gro* was downregulated, or of the remaining GFP-negative cells, is depicted as percentages in red and black, respectively. Note the increased number of cells at G1 phase following Gro downregulation. (C) Graph representing the number of GFP-positive cells per clone, in *lacZ*-expressing (left) as well as in *gro^MB36^*/+ cells expressing *gro* RNAi (right) clones, under the regulation of *hsflp;actin>CD2>nlsGFP* driver. RNAi-based reduction in Gro levels results in bigger clones, indicative of rapid cell cycles. *n*=number of clones analysed in each case. ***P<0.001 (Mann–Whitney U-test). In all cases, data represent the mean±s.d. (D) Schematic model depicting how phosphorylation and dephosphorylation of Gro during the cell cycle restrict its negative regulation of E2F1 to the S phase (see text for details). Scale bars: 100 µm (A); 16.6 µm (A′).

The derepression of E2F1 does not induce apoptosis of *gro*-deficient cells (Fig. S7), perhaps because the level of upregulated E2F1 in *gro* mutant cells is still below the threshold required to trigger cell death that is attained by E2F1 overexpression. To establish the significance of Gro-dependent repression at S phase, we determined how genetic depletion of *gro* affects the fate of eye imaginal disc cells posterior to the morphogenetic furrow. These cells are normally in S phase and, therefore, are EdU-positive (Fig. 3G-I) (Wolff and Ready, 1991). When homozygous mutant *gro^E48^* clones intersect this domain, cells in which E2F1 is derepressed and consequently upregulated are consistently EdU-negative, indicating that they are no longer in S phase (Fig. 7A,A′). The minority of *gro*-deficient cells that still stain for EdU, usually found at the margins of the clones, are typically E2F1-negative (Fig. 7A,A′; see Discussion).

We hypothesised that if Gro-mediated repression at S phase is crucial, then its absence might perturb proper cell cycle progression. To address this point, we profiled the cell cycle distribution of GFP-marked eye clones of cells heterozygous for the *gro^MB36^* null allele, in which *gro* levels were further knocked-down (*gro* RNAi) (Fig. S1). Flow cytometry analyses revealed that a greater proportion of GFP-positive cells with reduced *gro* levels have a 2N DNA content (Fig. 7B). Moreover, such eye imaginal disc clones show reduced staining for S, G2 and M phase markers (Fig. S7). They, therefore, accumulate predominantly at G1 phase.

We considered two possibilities that can explain this result: *gro* depletion either halts the cell cycle before DNA synthesis or accelerates the cell cycle such that cells pass through G2/M phases faster. To distinguish between these options, we determined the doubling times of clones of cells anterior to the morphogenetic furrow in eye imaginal discs overexpressing *lacZ* or, similarly, of cells in which RNAi was used to knock down *gro*, by quantifying the number of GFP-positive cells per clone (Fig. 7C). We found that the respective doubling time of *lacZ*-expressing control clones was, on average, 15.9 h as previously reported (Neufeld et al., 1998; Tseng and Hariharan, 2002). Strikingly, *gro* knockdown clones exhibit a significantly faster doubling time of 12 h. The accelerated cell cycle phenotype of cells with lower levels of *gro* is similar to that caused by E2F1 overexpression (Shibutani et al., 2008; Davidson and Duronio, 2012). A comparison between the average lengths of the different cell cycle stages in *gro* knockdown clones, relative to controls, revealed that the acceleration in the doubling time of the former stems from a shorter G2/M phase duration. Specifically, G1 phase in *lacZ*-expressing cells took 8 h, the duration of S phase was 2.44 h and G2/M phase was 5.3 h long. In *gro* RNAi cells, the corresponding times were 8.36 h, 2.15 h and 1.45 h, respectively. The higher proportion of cells at G1 phase in *gro* knockdown clones (Fig. 7B) resembled the accumulation of cells overexpressing E2F1 at G1 phase (Shibutani et al., 2008; Davidson and Duronio, 2012), supporting the notion that Gro is a negative regulator of *e2f1* at S phase.

In summary, our data indicate that Gro represses *e2f1* and possibly other genes at S phase. In the absence of Gro, the cycle accelerates and cells ultimately accumulate at G1 phase. Conversely, relief of Gro-mediated repression by Cdk1 activity is required for the correct progression through the G2 phase and for entry into mitosis (Fig. 7D). In light of the phenotypes caused by both depletion and overexpression of Gro, we conclude that Gro is a modulator of cell cycle regulation.

## DISCUSSION

Our results uncover a previously unrecognised tier of cell cycle regulation, and identify *e2f1* as a key target of Gro-mediated repression at S phase (Fig. 7D). Several observations point to a direct effect of Gro on *e2f1*. First, Gro is bound to chromatin within the *e2f1* gene locus in multiple cell lines and in *Drosophila* embryos (Fig. 6) (Orian et al., 2007; Roy et al., 2010; Kaul et al., 2014; Chambers et al., 2017). Second, Gro represses reporter gene expression derived from an *e2f1-lacZ* enhancer-trap line, which includes the sequences to which Gro binds (Fig. 6K-N) (Duronio et al., 1995; Brook et al., 1996). Third, E2F1 is repressed in cells that accumulate at G2 phase in response to ectopic Gro expression (Figs 5 and 6), despite the fact that it should be normally expressed at this stage (Shibutani et al., 2008; Davidson and Duronio, 2012). Thus, Gro negatively regulates E2F1, a transcription factor that functions at the heart of the cell cycle by activating multiple genes required for the initiation of the cycle, as well as for the G2 and M phases.

Previous reports have shown that preventing E2F1 proteolysis at S phase brings about accelerated cell cycles and/or apoptosis (Shibutani et al., 2008; Davidson and Duronio, 2012). Repression of *e2f1* by Gro does not appear to provide a robust, backup mechanism for preventing E2F1-induced apoptosis, because *gro* mutant cells are viable despite the upregulation of E2F1 (Figs 6, 7; Fig. S7). We surmise that elevated E2F1 protein levels in *gro*-deficient cells, in which the E2F1 degradation apparatus is presumably still operational, are not high enough to instigate cell death. Instead, E2F1 upregulation associated with the loss of Gro leads to accelerated cell cycles.

Surprisingly, a minority of *gro*-deficient cells do not upregulate E2F1 and still stain for EdU (Fig. 7A,A′). It is conceivable that these EdU-positive cells, usually found at the periphery of *gro* clones, are exposed to non-cell autonomous signals from surrounding cells that drive them into S phase, when E2F1 is robustly degraded. Their ability to continue dividing could explain why *gro* mutant clones are not eliminated.

Surprisingly, cells overexpressing Gro accumulate at G2 phase, and not at G1 phase as expected from depletion of *e2f1* (Dobrowolski et al., 1994; Ishizaki et al., 1996; Wu et al., 1996; Fan and Bertino, 1997; Dyson, 1998). We propose that overexpressed Gro blocks *de novo e2f1* transcription at G2 phase (following its repression by Gro at S phase) and, consequently, Gro aborts proper progression of the cycle at this stage. In contrast, the G1-to-S phase transition is refractory to Gro overexpression, as the E2F1 protein has already accumulated by then, and is post-transcriptionally regulated. Consistent with this interpretation, co-expression of E2F1 and Dp suppresses the G2-phase phenotype induced by Gro overexpression (Fig. 5L,L′).

Negative regulation by Gro is one of multiple layers of E2F1 control. Hence, the role of Gro appears to be modest in comparison with that of integral cell cycle regulators like Cdk1, which control diverse targets and processes during G2 phase and mitosis. Yet, E2F1 derepression alone cannot account for the overall outcome brought about by depletion of *gro*. Knockdown of *gro*, for example, leads to a shortened duration of the G2/M phases without affecting the length of the G1 phase, whereas overexpression of E2F1 causes shortening of both G1 and G2 phases (Neufeld et al., 1998). It is therefore conceivable that Gro also represses additional genes that must be silenced during S phase, in proximity to which it binds. One such prospective gene is *transforming acidic coiled coil* (*TACC*), the protein product of which is required to maintain spindle bipolarity and microtubule stability during mitosis (Trivedi, 2013). Silencing of *TACC* and/or other genes, besides E2F1, could also contribute to the predominant G2 phase arrest exerted by Gro expression (Fig. 5).

The full scope of Gro regulatory functions in the framework of the cell cycle will come to light once the complete repertoire of its targets is revealed and its DNA-binding partner proteins in this framework identified. How phosphorylated Gro is replaced by unphosphorylated Gro at S phase also remains an unanswered question. Future studies will further determine whether the accelerated cycles in *gro*-depleted cells lead to DNA damage sensitivity and/or to chromosomal aberrations.

The majority of pGro-positive cells are negative for pH3 staining and are, therefore, not mitotic (Fig. 1). These could be non-dividing cells or, if cycling, could be in one of the two Gap phases at which Gro is also phosphorylated (Fig. 2). Regardless, we propose that for cell division to occur, the attenuation of Gro repression must be accompanied by specific pro-proliferative cues. Such positive inputs, exclusive to this particular cellular process, will ensure that the downregulation of Gro by phosphorylation results in induction of a restricted sets of genes.

To date, Gro has been mainly implicated in transcriptional repression events controlling cell fate specification and differentiation (Hasson and Paroush, 2006; Cinnamon and Paroush, 2008). The new role we have uncovered for Gro and its phosphorylation in the cell cycle raises the possibility that it also functions in additional basic cellular processes. Accordingly, other physiological and/or metabolic processes, each employing a specific effector kinase(s), may induce their unique arrays of downstream target genes via phosphorylation of Gro.

Emerging evidence supports the notion that the Gro human TLE orthologues may act similarly to Gro in the context of the cell cycle. TLE1 and TLE3 are both implicated in cancer; TLE1, for example, promotes glioblastoma propagation (Dali et al., 2018) and TLE3 stimulates cell division by suppressing myogenic differentiation via transcriptional repression of the master regulator MyoD (Kokabu et al., 2017). Moreover, a large-scale analysis found that TLE1 is a mitotic bookmarking factor in development and in stem cells (Festuccia et al., 2017), and TLE3 was identified in a phosphoproteomics analysis of full phosphorylation site occupancy during mitosis (Olsen et al., 2010). Finding that TLE corepressors function comparably to Gro in cell cycle regulation may ultimately offer new therapeutic strategies for preventing uncontrolled cell division in cancerous settings.

## MATERIALS AND METHODS

### Fly culture and stocks

Flies were cultured and crossed on standard yeast-cornmeal-molasses-malt extract-agar medium at 25°C. cDNAs for Gro, Gro^AA^ and Gro^DD^ were cloned into the *pUAST-attB* vector, and all constructs were subsequently integrated at the *attp40* site (BestGene) to generate transgenic lines with comparable expression levels (Fig. S3) (Markstein et al., 2008).

The following GAL4 drivers and responders were used: *kr-Gal4* [Bloomington *Drosophila* Stock Center (BDSC), #58800]; *MS109*6-*Gal4*; *gro^MB36^*, *UAS-gro RNAi*/*TM6B* (generously provided by Gerardo Jiménez, Institut de Biologia Molecular de Barcelona-CSIC, Barcelona, Spain) (Jennings et al., 2008); *UAS-cdk1 RNAi* (BDSC, #28368 and #36117); *UAS-cdk2 RNAi* (BDSC, #28952 and #34856); *UAS-cdk4 RNAi* (BDSC, #36060 and #27714); *UAS*-*LacZ* (Brand and Perrimon, 1994); *UAS-GFP* (Yeh et al., 1995); *UAS*-*E2F1, UAS*-*Dp/CyO* (BDSC, #4774); *PCNA-GFP* (kind gift of Robert Duronio, University of North Carolina, Chapel Hill, USA); *P[rm729] e2f1-lacZ* (BDSC, #34054); and *Fly-FUCCI* (BDSC, #55124 and #55123) (Zielke et al., 2014). *yellow white* flies served as *wild-type* controls.

### Generating *groucho* loss-of-function and overexpression clones

Mutant clones lacking functional Gro (*gro^E48^*) were generated using FLP-mediated mitotic recombination in the progeny of the following cross: *hsflp*; *P[FRT82B] ubi-GFP/TM6B* virgin females and *P[FRT82B] gro^E48^/TM6B* males. Clones were induced 48-72 h after egg laying by heat-shock (60 min at 37°C) and were identified by the loss of the GFP marker and the concurrent appearance of a twin spot clone. Knockdown of *gro* was attained by crossing *hsflp*; *actin>CD2>Gal4*; *UAS-nlsGFP/TM6B* virgins to *gro^MB36^*, *UAS-gro RNAi*/*TM6B* males (Jennings et al., 2008). Clones, induced 48-72 h after egg laying by heat-shock (10 min at 37°C), were distinguishable via the GFP marker.

### Western blotting

Wing imaginal disc lysates were prepared for immunoblotting as previously described (Kushnir et al., 2020). Western blotting was carried out using a standard protocol. Briefly, samples were separated on SDS-PAGE (10 cm × 10 cm) and proteins were electro-transferred at 100 mV for 90 min to 0.45 µm Nitrocellulose membranes (Whatman). Membranes were washed with Tris-buffered saline containing 0.1% Tween 20 detergent (TBST) for 5 min at room temperature and incubated with blocking buffer [1× TBS, 0.1% Tween-20 with 5% w/v nonfat dry milk or 5% bovine serum albumin (BSA)] for 1 h at room temperature. Membranes were subsequently washed three times for 5 min in TBST, and incubated in buffer containing the primary antibody in buffer (1× TBS, 0.1% Tween-20 with 5% BSA) overnight at 4°C. Membranes were then washed three times for 5 min with TBST, and incubated with the appropriate HRP-conjugated secondary antibody in buffer (1× TBS, 0.1% Tween-20 with 1% BSA). After three washes for 5 min with TBST, proteins were detected using Pierce ECL Western Blotting Substrate (ThermoFisher Scientific) in accordance with the manufacturer's instructions.

### Antibody staining

Primary antibodies used in this study were: rabbit anti-phospho-Histone 3 (1:100; Cell Signaling Technology, #9701); mouse anti-phospho-Histone 3 (1:100; Cell Signaling Technology, #9706); rabbit anti-pGro (1:100; Hasson et al., 2005; Cinnamon et al., 2008); mouse anti-Gro (diluted 1:1000 for immunofluorescence and 1:5000 for western blot analysis; generously contributed by Christos Delidakis, Institute of Molecular Biology and Biotechnology, Crete, Greece) (Delidakis et al., 1991); rat anti-total Gro (1:1000 for western blot analysis; Santa Cruz Biotechnology, sc-15786); rabbit anti-panTLE (1:100; Cell Signaling Technology, #4681); mouse anti-GFP [1:100; Developmental Studies Hybridoma Bank (DSHB), #8H11]; mouse anti-Cyclin A (1:20; DSHB, #A12); mouse anti-Cyclin B (1:20; DSHB, #F2F4); rabbit anti-Dcp1 (1:100; Cell Signaling Technology, #9578); rat anti-E2F1 (1:100; generously contributed by Stefan Thor, University of Queensland, Australia); and rabbit anti-HA (1:100; Cell Signaling Technology, #3724). Secondary antibodies were Alexa Fluor 488 AffiniPure Donkey Anti-Mouse IgG (1:400; Jackson ImmunoResearch 715-545-150), Rhodamine Red-X (RRX) AffiniPure Donkey Anti-Mouse IgG (1:400; Jackson ImmunoResearch 715-295-150), Cy5 AffiniPure Donkey Anti-Mouse IgG (1:400; Jackson ImmunoResearch 715-175-151) or Cy5 AffiniPure Goat Anti-Rabbit IgG (1:400; Jackson ImmunoResearch 111-175-144).

Nuclei were labelled using 4′,6-diamidino-2-phenylindole (DAPI) (1:1000; Sigma-Aldrich), and embryos and wing imaginal discs were mounted using Vectashield medium (Vector Laboratories).

In all cases, 90-100 imaginal discs for each genetic background were subjected to immunofluorescent antibody staining. Each staining was repeated at least three independent times.

### Immunovisualisation of Gro phosphorylation state *in vivo*

Rabbit anti-phosphorylated-Gro (pGro) polyclonal antibodies were raised using a synthetic phosphopeptide containing one of two Erk consensus sites (phospho-Threonine 308), which is highly conserved in different *Drosophila* species as well as in other insects (Fig. S2). Subsequently, the antibodies were affinity-purified on a column with a corresponding non-phosphorylated peptide, and the flow-through was later bound on a column with the phosphorylated peptide. These anti-pGro antibodies detect Gro in its phosphorylated state *in vivo*, particularly in domains of ongoing and earlier RTK pathway activity (given the persistence of Gro phosphorylation) (Hasson et al., 2005; Cinnamon et al., 2008; Helman et al., 2011; Johnston et al., 2016).

When diluted up to 1:100, the monoclonal mouse anti-Gro antibody, raised against amino acids 120-380 (a region containing the epitope used to generate the rabbit poly-clonal anti-pGro antisera) (Delidakis et al., 1991), detects Gro whether phosphorylated or not (Delidakis et al., 1991). When used at a 1:1000 dilution, however, this antibody primarily recognises non-phosphorylated Gro, generating a signal that largely complements the domain of pGro throughout embryonic and adult development (Fig. S1) (Cinnamon et al., 2008; Johnston et al., 2016). Note that the anti-Gro antibody detects signals across a range of Gro protein levels (i.e. downregulation as well as overexpression; Fig. S1 and Fig. 4). The general lack of anti-Gro staining in anti-pGro-positive nuclei (Fig. 1A,A′) implies that the protein is phosphorylated in these nuclei. Importantly, the mutually exclusive recognition by the anti-pGro and anti-Gro antibodies is also observed *in vitro* even under denaturing conditions (Cinnamon et al., 2008), suggesting that both antibodies are probably directed against the same epitope and that phosphorylation is enough to mask detection by the anti-Gro antibody.

The specific recognition of nonphosphorylated Gro by the anti-Gro antibody is further illustrated by its failure to detect phosphomimetic Gro^DD^, while strongly recognising the nonphosphorylatable Gro^AA^ variant when similarly overexpressed (see insets in Fig. 4C and A, respectively). Finally, commercially available polyclonal anti-total Gro antibodies (Santa Cruz Biotechnology) were used to detect total Gro levels (i.e. Gro, Gro^AA^ and Gro^DD^) in immunoblots (Fig. S3), and commercially available anti-panTLE antibodies (Cell Signaling Technology) were used to follow transgenic expression *in vivo* (Fig. S3).

### EdU incorporation

Third instar larval wing and eye imaginal discs were submerged in 1× PBS in the presence of 1:1000 EdU for 1 h with gentle rolling at room temperature. EdU was detected using the Click-iT EdU Alexa Fluor 555 Imaging Kit (Life Technologies).

### *In vitro* kinase assay

A GST-tagged, full-length Gro fusion protein was expressed in *Escherichia coli* and purified on a Glutathione affinity column. Approximately 2 µg of purified protein were incubated with (or without) an activated Cdk1/CyclinB1 complex (Sigma-Aldrich, SRP5009) as per the manufacturer's instructions, with the following changes: the final reaction conditions were 10 mM MOPS (pH 7.2), 5 mM glycerol 2-phosphate, 10 mM MgCl_2_, 2 mM EGTA, 0.8 mM EDTA, 0.1 mM dithiothreitol (DTT) and 50 μM ATP. The reactions were proceeded for 30 min at 30°C and terminated by adding sample buffer. Reaction mixtures were separated on SDS-PAGE and analysed by western blotting with anti-pGro and anti-Gro antibodies.

### Real-time polymerase chain reaction

Total RNA was extracted using Aurum Total RNA Mini Kit (Bio-Rad). RT-PCR was performed using iTaq Universal SYBR Green Master Supermix (Bio-Rad) and CFX384 Touch Real-Time PCR Detection System (Bio-Rad). Expression was normalised to *GAPDH* transcripts in all cases. Each experiment was carried out in biological triplicates, with three technical replicates measured each time (one representative experiment is shown). Data analysis was preformed using Bio-Rad CFX manager 3.1 (Bio-Rad).

### Flow cytometry

Samples were prepared as previously described (Davidson and Duronio, 2012), and DNA content was determined using Hoechst 33342 (Sigma-Aldrich). Cells were subjected to flow cytometry using LSR-Fortessa Analyzer (BD Biosciences), and results were analysed by FCS express 4 software (De-Novo Software). Each experiment was carried out at least three independent times, of which a single representative experiment is presented. In all experiments, larvae were identically staged based on hours after egg laying; the GFP population gated separately and afterwards overlaid; and an equal number of cells per each population are presented.

### Quantifying mitotic indices and doubling time of cell populations

Mitotic indices, which take into account the effect of Gro on mitosis while controlling for the changes its expression exerts on cell size, were calculated by counting pH3-positive cells in a known, constant area and dividing them by the number of nuclei.

For doubling time measurement, clones were induced by heat shock at 72 h and fixed at 124 h. Doubling time was calculated using the following formula: (Log 2/Log N)h, where N is the median cell number per clone and h is the age of the clones in hours (Tseng and Hariharan, 2002).

### Semi-automated calculation of overlapping signals

We used the ImageJ ROI manager to semi-automatically calculate the percentage of overlapping area (in pixels) between two different signals. Each colour threshold was automatically determined, and individual mask selections were constructed. The ‘AND’ tool in the ROI manager was then used to determine the area of overlap between the two masks, and the ‘OR’ tool to determine the area covered by both masks. The percentages presented in the Figures represent the division of the ‘AND’/‘OR’ values.

### Calculating the length of the different cell cycle phases

The doubling time in each genotype was first determined, and the distribution (i.e. percentage) of cells at each phase of the cell cycle was then established using flow cytometry (Neufeld et al., 1998).

### ChIP-seq

ChIP-seq assays were carried out for Gro binding in Kc167 and S2R+ cells as previously described (Kaul et al., 2014). Two biological ChIP-seq replicates were used to obtain a high confidence set of peaks for Gro in BG3 cells. Peaks were called using the MACS2 v2.1.1.2 software providing input and immunoprecipitated samples simultaneously with default parameters. Peaks present in both biological replicates, with FDR<10% in at least one sample and *P*-value<0.001 in the other, were selected (Gaspar, 2018 preprint).

Sequences were aligned to the dm6.26 reference genome using bowtie2 v2.3.3 according to default parameters (Langmead and Salzberg, 2012). Unmapped reads were removed and adapters were trimmed. Multimapping reads were removed using the ‘view’ program of SAMtools v1.7 (Li et al., 2009) with the parameter ‘-q 20’. The program ‘markdup’ was used to remove PCR duplicates from mapped reads, with the parameter ‘-r’.

BigWig files were generated from BAM files using bamCoverage from deepTools v3.4.3, and reads normalised to counts per million (CPM) were mapped. BigwigCompare was then used to normalise each file against its input using a bin size of 10 and operation subtract as parameters. Visualisation of the genome tracks of ChIP-seq signals was attained using the Integrate genomics viewer (Thorvaldsdóttir et al., 2013).

ChIP-seq datasets for Gro in Kc167 and S2R+ cells have been previously published (Kaul et al., 2014) and are available from ArrayExpress (E-MTAB-2316). The accession number for the Illumina Sequencing data for Gro ChIP-seq in BG3 cells from this study is ArrayExpress (E-MTAB-12108).

### Microscopy

Adult wings were mounted as previously described in Kushnir et al. (2020). Confocal images were attained using a Zeiss LSM710 confocal microscopy. Images were processed using Adobe Photoshop software.

### Statistical analyses

Statistical analyses (binomial, two-tailed and Mann-Whitney U tests) were conducted using GraphPad Prism 8 software. ImageJ was used to measure the intensity of yellow-stained cells and for semi-automated quantifications, and Zen software was used to quantify the numbers of DAPI, pH3-, EdU-, Gro- or pGro-positive cells.

## Supplementary Material

10.1242/develop.201041_sup1 Supplementary information
