# Supplementary material for: Normal cell cycle progression requires negative regulation of E2F1 by Groucho during S phase and its relief at G2 phase
Source: Development. 2023 Jun 1;150(11):dev201041. doi: 10.1242/dev.201041 (PMC10281550; doi:10.1242/dev.201041)
Supplement: Supplementary information [file develop-150-201041-s1.pdf]

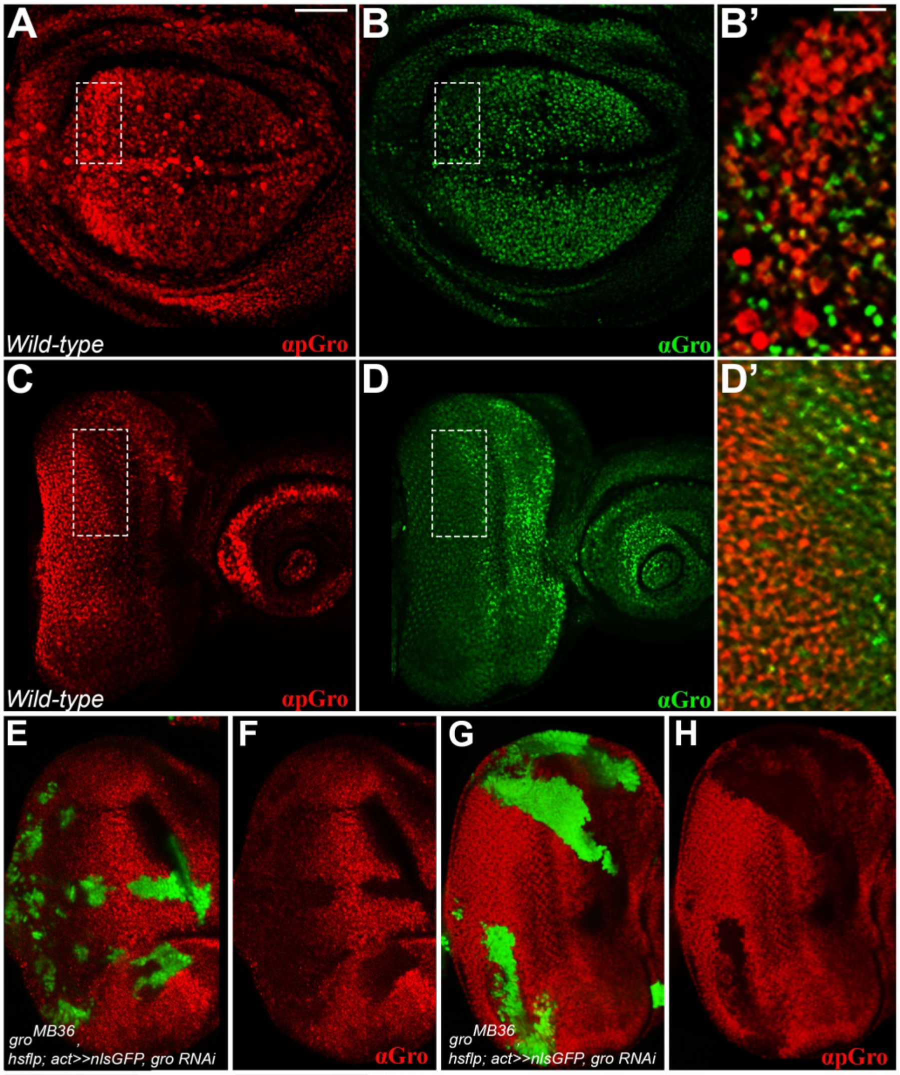

Fig. S1. Immunovisualisation of Groucho's phosphorylation state *in vivo* using anti-Gro and anti-phospho-Gro antibodies. (A-D') Confocal images of *wild-type* third instar wandering larval wing (A-B') and eye (C-D') imaginal discs, co-stained for pGro (red; A, B', C, D') and Gro (green; B-B', D-D'). (B', D') Magnified views of boxed regions in (A-B and C-D), respectively. The general complementarity in epitope detection by the anti-pGro and anti-Gro antibodies is evident (Cinnamon et al., 2008; Johnston et al., 2016). The relative prevalence of anti-pGro staining compared to that of anti-Gro probably stems from the persistence of Gro phosphorylation (Helman et al., 2011). (E-H) Both the anti-Gro antibody and the anti-pGro antibodies are sensitive to RNAi-mediated reduction in Gro levels. Eye imaginal discs, in which *gro* was knocked-down in clones of cells heterozygous for the *gro*^{MB36} allele (discernable by GFP staining; green; E, G), were stained for Gro (red; E-F) or for pGro (red; G-H). Note the reduced intensity of the anti-Gro (E-F) and anti-pGro (G-H) signals in the clones. Scale bar = 100 µm (A-B, C-D, E-H) and 33.33 µm (B', D').

Development • Supplementary information

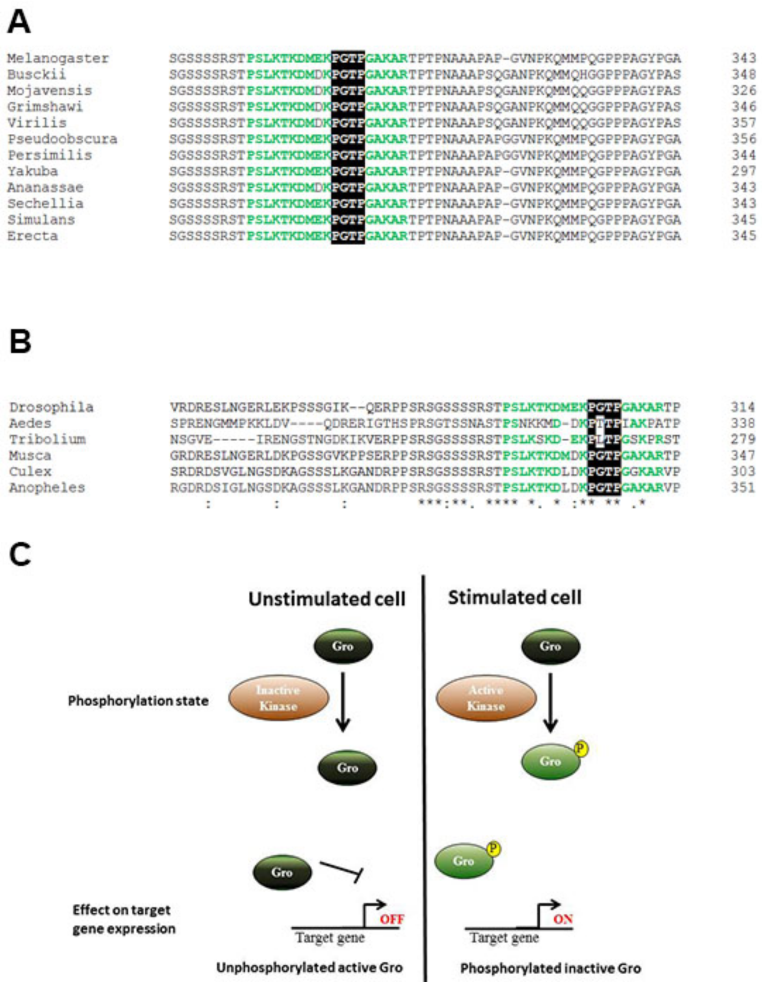

Fig. S2. Groucho's Erk/Cdk1 phosphorylation site is highly conserved. (A) Amino acid alignment, showing that the consensus Erk/Cdk1 phosphorylation site (black) is fully conserved in Gro orthologs from 12 *Drosophila* species. Note that the sequence used to immunize rabbits for generating the anti-pGro antibodies, demarcated in green, is also highly conserved. (B) The Erk/Cdk1 phosphorylation motif (black) and the amino acid sequence used to generate the anti-pGro antibodies (green) are also conserved to a high degree in Gro orthologs from other insects. (C) Model portraying relief of Gro-mediated repression by phosphorylation.

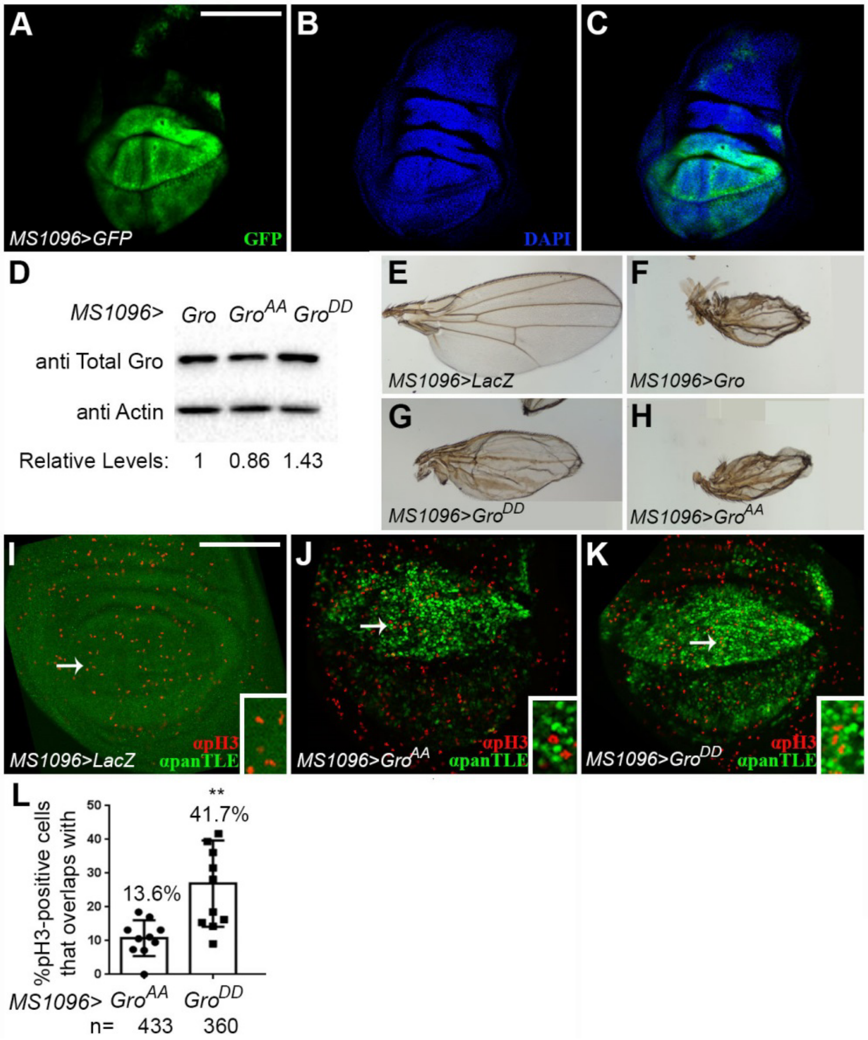

Fig. S3. Transgenic expression of Gro, Gro^{AA} and Gro^{DD} using the *MS1096-Gal4* driver. (A-C) Confocal image of third instar wandering larval wing imaginal disc, in which *MS1096-Gal4* drives expression of GFP (green; A, C), counterstained for 4',6-diamidino-2-phenylindole (DAPI) (blue; B-C). GFP is expressed predominantly in the dorsal region of the wing imaginal disc, but also in many cells in the ventral compartment (albeit to a lesser extent), probably due to leakiness of the *MS1096-Gal4* driver. Note the uneven, irregular nature of the UAS/Gal4 overexpression system.

(D) Immunoblot analysis showing that relative transgenic expression levels of Gro, Gro^{AA} and Gro^{DD}, driven by *MS1096-Gal4*, are comparable. Relative levels of Gro^{AA} and Gro^{DD}, determined based on the ratio between total Gro and Actin levels, were normalized to these values for Gro. The immunoblotting was repeated 3 independent times.

(E-H) Wings of adult females of the indicated phenotypes.

(I-K) Confocal images of third instar wandering larval wing imaginal discs, overexpressing LacZ (I), Gro^{AA} (J) or Gro^{DD} (K) under the regulation of the *MS1096-Gal4* driver, co-stained for panTLE (green) and pH3 (red). Insets in (I-K) show magnified views of regions marked by

Development • Supplementary information

respective arrows. Note that anti-panTLE antibodies, which are insensitive to Gro's phosphorylation state and therefore recognize both Gro^{AA} and Gro^{DD}, weakly detect endogenous Gro (I) but visibly detect overexpressed Gro^{AA} (J) and Gro^{DD} (K). (L) A significantly larger proportion of pH3-positive mitotic cells overlaps with Gro^{DD} than with Gro^{AA}. Each dot in the graph represents the relative percentage of pH3-positive mitotic cells that overlap with staining for Gro^{AA} or for Gro^{DD} in a single wing imaginal disc (9 discs were analyzed for Gro^{AA} and 11 for Gro^{DD}). The numbers above the graph denote the average percentage of pH3 cells that overlap with the Gro^{AA} or Gro^{DD} signals, respectively. n = number of pH3-positive cells scored in each case. ** $P < 0.01$ (*Mann–Whitney U*-test). Data represents the mean ± SD. Scale bars = 200 µm (A-C) and 100 µm (I-K).

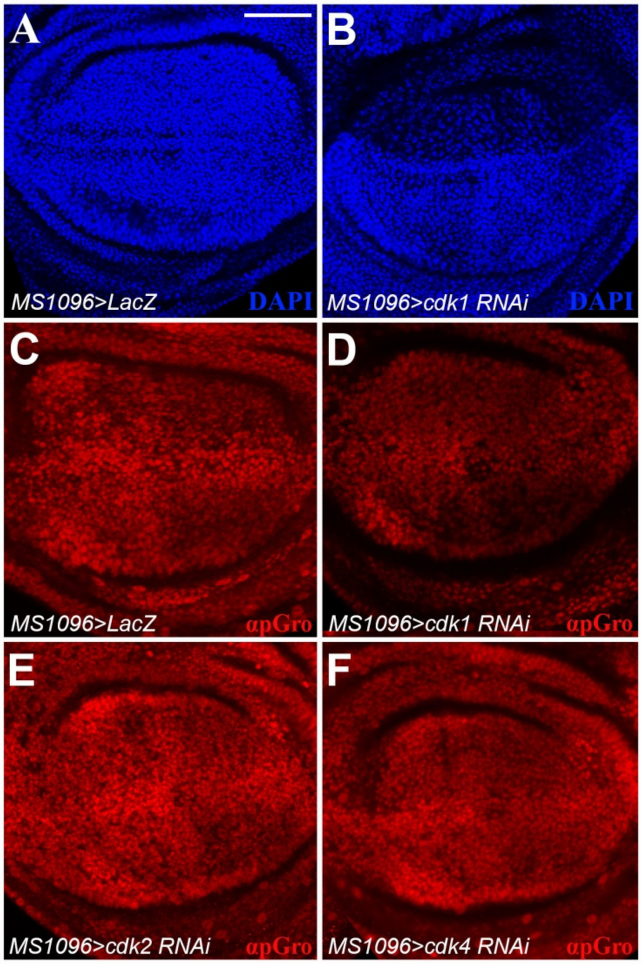

Fig. S4. RNA interference-based reduction in Cdk1 levels results in fewer and larger cells, as well as in decreased levels of phosphorylated Groucho. (A-B) Confocal images of third instar wandering larval wing imaginal discs, expressing either LacZ (A) or an RNA interference (RNAi) construct for *cdk1* (B), stained with DAPI (blue). Note that fewer and larger nuclei are observed in the domain of *cdk1* downregulation, as previously reported (Bettencourt-Dias et al., 2004; Johnston, 1998) . (C-F) *RNAi*-based knockdown of *cdk1* (D), but not of *cdk2* (E) or *cdk4* (F), leads to a ~15% decline in phosphorylated Gro (*cf.* LacZ-expressing disc; C). Due to high variability, however, this decrease is statistically non-significant (p=0.2854; *Mann–Whitney U*-test). Similar results were observed in wing imaginal discs in which the *en-Gal4* driver was used to express *cdk1 RNAi*. Scale bar = 100 µm (A-F).

Development • Supplementary information

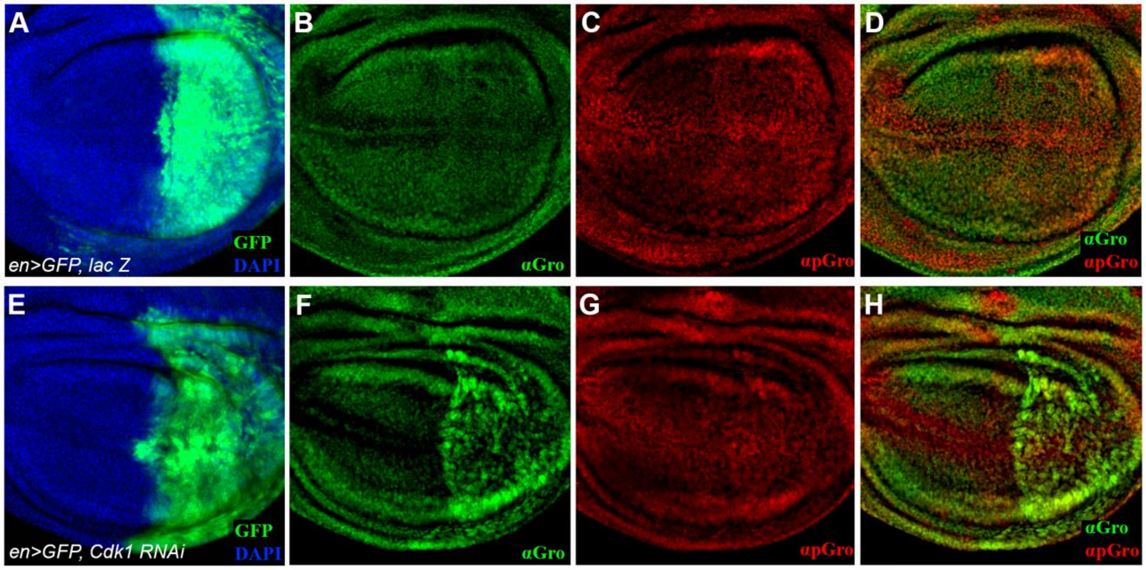

Fig. S5. Reduced levels of phosphorylated Groucho in *en*>GFP, *cdk1 RNAi*-expressing wing imaginal discs. (A-H) Confocal images of third instar wandering larval wing imaginal discs, expressing either LacZ (control; A-D) or an RNA interference (RNAi) construct for *cdk1* (E-H), under the *en-Gal4* driver in the posterior compartment (demarcated by GFP; green; A, E). Discs were co-stained for Gro (green; B, D, F, H) and pGro (red; C, D, G, H), and nuclei were marked by DAPI (blue; A, E). Note the relative increase in the anti-Gro signal upon Cdk1 knockdown (F, H; 1.686±0.8979) in comparison to control (B, D; 0.9098±0.09922) (p<0.0001; *Mann–Whitney U*-test). The relative pGro signal is lower in the posterior compartment of Cdk1 knockdown discs (G; 0.7267 ±0.2299) compared to controls (C; 1.269±0.1285) (p<0.0001; *Mann–Whitney U*-test). The relative pGro levels were compared to those of Gro in the anterior and posterior compartments separately, and the ratios normalized for each individual disc (posterior/anterior). Values were then compared between the two backgrounds (10 discs for each genotype) using the *Mann–Whitney U*-test.

Development • Supplementary information

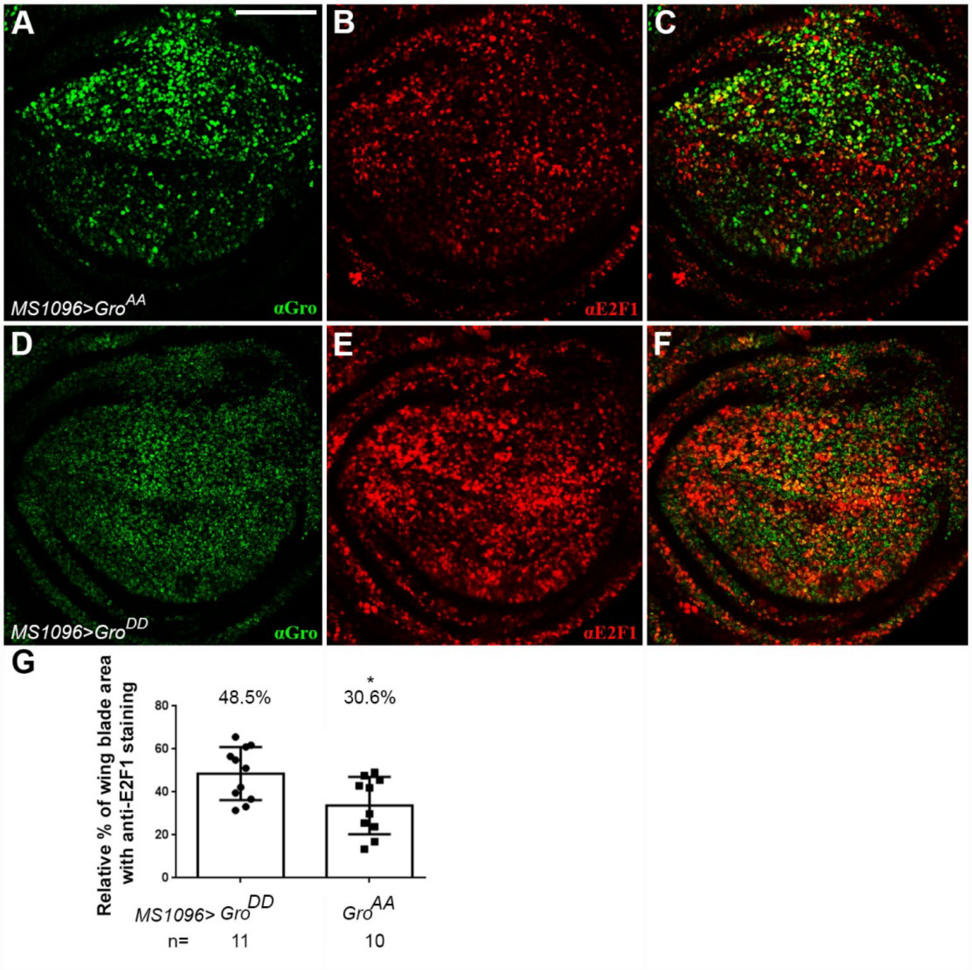

Fig. S6. Gro^{AA}, but not Gro^{DD}, represses *e2f1* expression. (A-F) Confocal images of third instar wandering larval wing imaginal discs overexpressing Gro^{AA} (A-C) or Gro^{DD} (D-F) under the regulation of the *MS1096-Gal4* driver, co-stained for Gro (green; A, C, D, F) and E2F1 (red; B-C, E-F). Note the overall decrease in anti-E2F1 staining in the Gro^{AA}-expressing disc (B), in comparison to the disc expressing Gro^{DD} (E). (G) Graph showing relative percentage of area covered by anti-E2F1 staining in the wing blade in each genotype. Each dot in the graph represents the relative percentage measured in a single wing imaginal disc (11 discs were analyzed for Gro^{DD} and 10 for Gro^{AA}). The numbers above the graph designate the average percentage of wing blade area stained for E2F1 in Gro^{AA} or Gro^{DD} wing imaginal discs, respectively. * $P < 0.05$ (*Mann–Whitney U*-test). Data represents the mean ± SD. Scale bar = 100 µm (A-F).

Development • Supplementary information

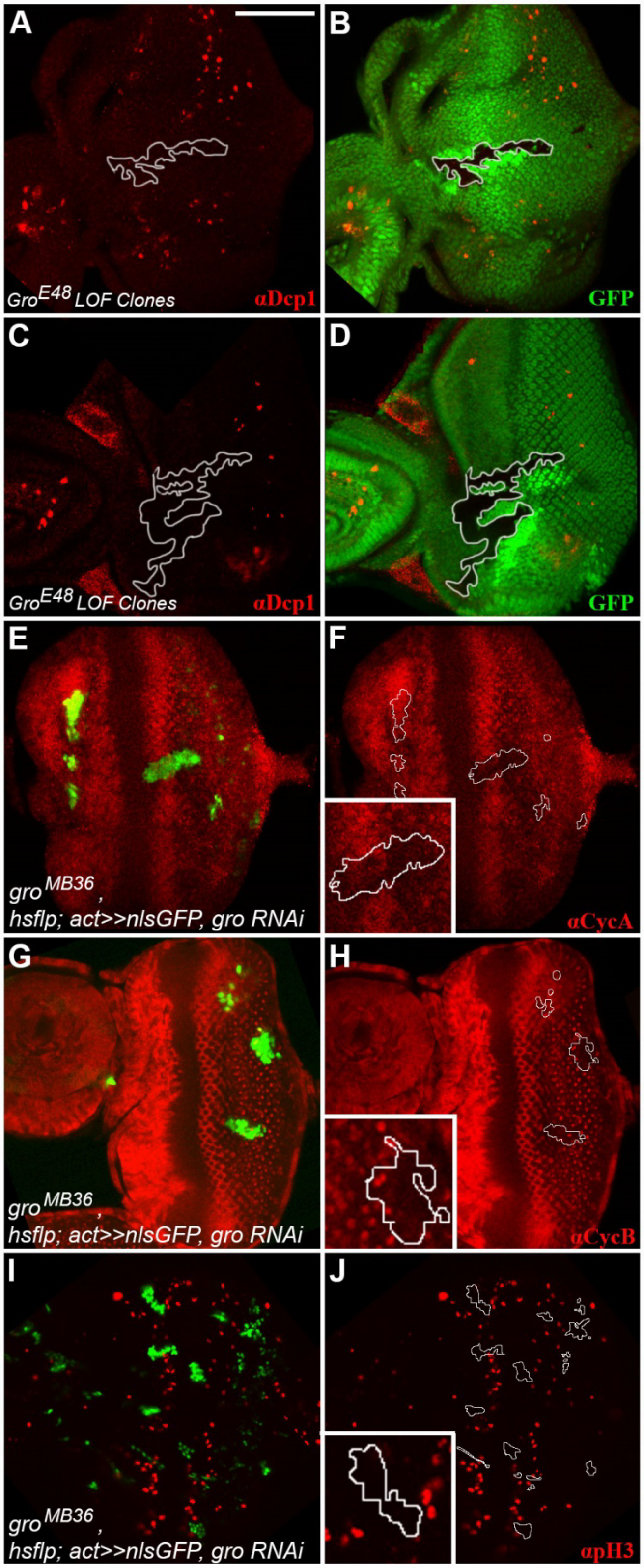

Fig. S7. Cells with reduced Groucho levels neither undergo apoptosis nor stain for S-, G2- and M-phase markers. (A-D) GFP-negative (green; B, D) homozygous *gro*^{E48} loss-of-function clones (demarcated by white contours), induced in larval eye imaginal discs, stained for the activated

Development • Supplementary information

form of the *Drosophila* effector caspase, *Drosophila* caspase 1 (Dcp-1) (red; A-D). Anti-Dcp-1 staining is not elevated in *gro* clones and their overall size is similar to their respective twin clones.

(E-J) Confocal images of third instar wandering larval eye imaginal discs, in which *gro* was knocked-down in GFP-labelled clones of cells heterozygous for the gro^{MB36} allele (green; E, G, I). (F, H, J) Clonal boundaries are outlined, with each inset showing a magnified view of a representative clone. Note that most cells, in which *gro* levels are reduced, do not stain for the S- and G2-phase marker CycA (red; E-F); for the G2-phase marker CycB (red; G-H); or for the mitotic marker pH3 (red; I-J). Scale bar = 100 µm.

Table S1. List of candidate Gro-repressed targets that emerged from an unbiased *in silico* analysis, and the number of lists they appear in (see text for details).

Gene name	Number of lists
InR	5
fz	5
pros	5
e2f1	4
N	4
ptp61F	4
enc	4
esg	4
fz2	4
insc	4
tacc	4
ci	3
egfr	3
vg	3
wg	3
galphai	3
ser	3
hh	3
pan	3
aPKC	3
EcR	3
hsp83	3
wts	3
jra	3
Rho1	3
stg	3
src64B	3
cnn	3
ex	3
S	3

Development • Supplementary information

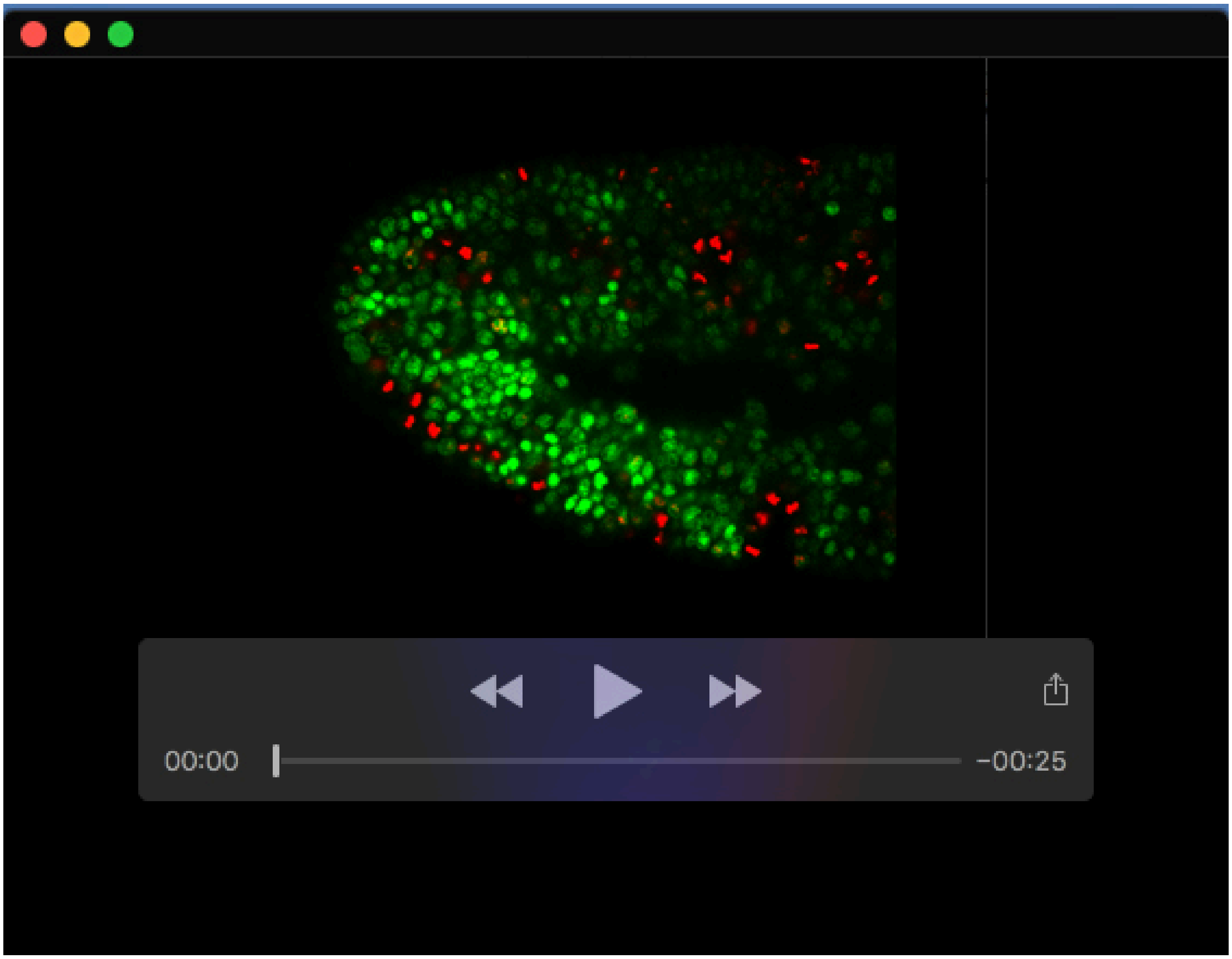

Movie 1. Z-stack imaging of a stage 11 *Kr>Gro* embryo, co-stained for pH3 (red) and Gro (green). Note that the two signals are largely non-overlapping.
